# Efficient Organic Molecular Crystal Structure Prediction Using the Density Functional Tight-Binding Method

**DOI:** 10.1021/acsphyschemau.6c00036

**Published:** 2026-06-20

**Authors:** Maureen M. Kitheka, Yan Jing, Yan Yao, Puja Goyal

**Affiliations:** a Department of Chemistry, 14787 State University of New York at Binghamton, Binghamton, New York 13902, United States; b Department of Electrical and Computer Engineering and Texas Center for Superconductivity (TcSUH), 14743 University of Houston, Houston, Texas 77204, United States; c Department of Chemistry, 6049 Illinois State University, Normal, Illinois 61761, United States

**Keywords:** crystal structure prediction, particle swarm optimization, CALYPSO, density-functional tight binding, DFTB, pyrene-4,5,9,10-tetrone

## Abstract

Over the years, computational crystal structure prediction (CSP) for organic molecules has thrived as an area of research, spanning various scientific disciplines and having significant applications in industries such as pharmaceuticals and agrochemicals. Within the field of batteries, redox-active organic materials (ROMs) such as quinones have received increased attention as promising electrode materials for rechargeable batteries. However, experimental determination of the crystal structure of intermediate species formed during the discharge/charge cycle can often be challenging. Incomplete X-ray diffraction patterns can also lead to difficulties in crystal structure determination for ROMs used in batteries. Use of a semiempirical electronic structure method for CSP helps to avoid force field reparameterization for different species, sometimes with complex electronic structure, formed during battery operation. It also helps to significantly lower the computational cost compared to the widely used density functional theory (DFT). In this study, we report the success of a CSP algorithm based on a combination of the particle swarm optimization method, as implemented in the CALYPSO software, with third-order density functional tight-binding, a DFT-based semiempirical method. Accompanied by data postprocessing using DFT, this method enables the correct identification of the most stable crystal structures of organic molecules with different kinds of intermolecular interactions ranging from hydrogen bonding to π-stacking. We also report the experimental crystal structure of pyrene-4,5,9,10-tetrone, a molecule studied intensively for application in organic batteries, and predict its crystal structure correctly using our method. Our findings emphasize the potential of this approach for CSP of different classes of organic molecules, including quinones. Additionally, they establish the foundation for future CSP studies of other organic molecules utilized in rechargeable batteries.

## Graphical abstract

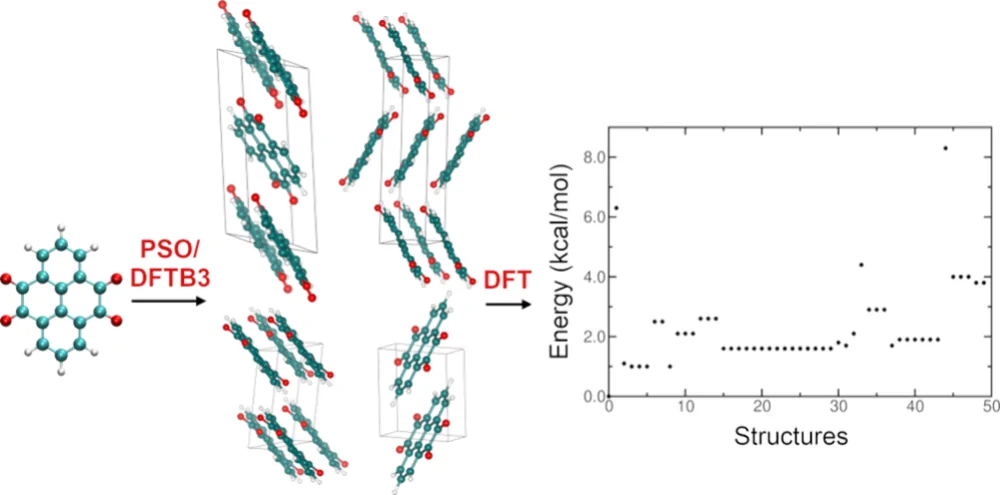

## Introduction

Crystal structure prediction (CSP) for organic molecules has been a topic of great interest to the computational chemistry community for several decades.^1^ It has special relevance to the pharmaceutical industry, which is continually interested in more stable polymorphs of a drug candidate molecule.^2–4^ Organic molecules are finding an increasingly important place in the field of batteries, with several research groups in search of organic materials as replacement for inorganic electrode materials.^5–8^ Crystalline quinones have been quite extensively investigated as alternatives to inorganic electrode materials in rechargeable lead-acid batteries,^9^ lithium-ion batteries,^10^ and magnesium ion batteries,^11^ just to name a few. Very often, experimental determination of the crystal structure of intermediate species formed during battery operation is cumbersome. In addition, X-ray diffraction patterns can sometimes be incomplete, leading to the difficulty of experimental determination of the crystal structures of organic molecules like quinones used in batteries.

Several blind tests for CSP over the years have revealed the strengths and weaknesses of the myriad of computational methods utilized for CSP.^12^ These methods involve a candidate structure generation algorithm and a method for structure optimization/energy determination. The particle swarm optimization (PSO) algorithm^13,14^ implemented in the CALYPSO software package^15,16^ has found immense success in predicting the crystal structure of inorganic materials under extreme temperature and pressure conditions. It has, however, not been reported to be applied to organic molecules. PSO or any candidate structure generation algorithm can be used in conjunction with any method for structure optimization/energy determination. Because of the variable redox states of organic materials during battery operation, the use of a classical force field for structure optimization/energy determination is not desirable. Density functional theory (DFT) is widely used in CSP. Its computational expense is, however, not negligible. As highlighted in our previous study,^17^ a computationally efficient yet accurate method for modeling organic electrode materials in batteries is highly desirable. Being 2-3 orders of magnitude faster than DFT, the density functional tight-binding (DFTB)^18^ method is a very promising option in this regard. Currently, the method allows the inclusion of up to third order terms in the Taylor series expansion for the DFT energy (DFTB3) as expressed in eq 1.^19^

$$E^{\mathrm{DFTB}3}=\frac{1}{2}\underset{ab}{\sum }V_{ab}^{rep}+\underset{iab}{\sum }\underset{\mu \epsilon a}{\sum }\underset{\nu \epsilon b}{\sum }n_{i}c_{\mu }^{i}c_{\nu }^{i}H_{\mu \nu }^{0}+\frac{1}{2}\underset{ab}{\sum }\gamma_{ab}^{h}{\Delta q}_{a}{\Delta q}_{b}+\frac{1}{3}\underset{ab}{\sum }\Gamma_{ab}\Delta q_{a}^{2}\Delta q_{b}$$ 1

where *V*_*ab*_^*rep*^ represents the repulsive potential, *n*_*i*_ denotes the occupation number of Kohn–Sham orbital *i*, *c*_μ/ν_^*i*^ are the molecular orbital coefficients, *H*_*μν*_^0^ are the Hamiltonian matrix elements based on the reference density, γ_*ab*_^*h*^ is the Coulomb interaction between atoms a/b, Δ*q*_*a*/*b*_ are the Mulliken charges on atoms a/b, and Γ*_ab_* is the charge-dependent Hubbard derivative parameter.

Brandenburg et al.^20^ have reported the potential of DFTB3/3OB^21^ with the D3 ^22^ dispersion correction to describe the noncovalent interactions in organic molecular crystals, yielding a mean absolute deviation of only 2.5 kcal/mol in the lattice energies for the X23 data set.^23,24^ Subsequently, the same authors^25^ tested DFTB3-D3 for equilibrium geometries, energy ranking, and stability analysis of their benchmark set, POLY59, and found it to overestimate crystal mass densities. However, CSP using DFTB3-D3 was not carried out in these studies.

Mortazavi et al.^26^ combined DFTB3/3OB with a variety of dispersion correction methods, and similar to Brandenburg et al., found it to underestimate unit cell volumes for the X23 data set significantly. Relative energies of low energy polymorphs of coumarin and a molecule from the sixth CSP blind test were found to be described well if PBE+vdW single point calculations were carried out on DFTB3+vdW structures. The authors proposed that DFTB3+vdW can be used as a prescreening tool in CSP and can be followed up by DFT+vdW single point calculations on DFTB3+vdW structures for accurate ranking of polymorphs.

Wengert et al.^27,28^ recently demonstrated that machine learning (ML)-based corrections to DFTB+TS, where TS represents the Tkatchenko-Scheffler dispersion correction,^29^ lead to a Δ-ML model which is capable of describing lattice energies and energy ranking of organic crystals with comparable accuracy to PBE+MBD, where MBD^30,31^ represents many body dispersion correction. Their study suggests that in a CSP study, using DFTB+TS for the initial screening of the large set of candidate structures or/and the ranking of a final set of favored candidates in order of energy would lead to erroneous results. However, their study did not carry out a full-fledged CSP study for any molecule using DFTB+TS, PBE+MBD, or the Δ-ML model.

To the best of our knowledge, the only publicly reported CSP study using DFTB was carried out by Iuzzolino et al.^32^ They focused on CSP for molecules relevant to the pharmaceutical industry. DFTB3-D3 was found to have similar accuracy to PBE0 partial optimization for reproducing the experimental conformers, but it could not provide an accurate energy ranking of different crystal structures. This led to the necessity of reoptimizing selected DFTB3-D3 structures with a DFT-based method.

In this study, we carry out a systematic examination of the ability of DFTB3 to predict the most stable crystal structures of different classes of organic molecular crystals. We employ the PSO algorithm implemented in CALYPSO in conjunction with the DFTB method implemented in the DFTB+ software package to sample low energy crystal structures of organic molecules with different classes of intermolecular interactions, followed by reoptimization of the 50 lowest energy crystal structures with DFT (PBE). Finally, we apply this approach to the CSP of pyrene-4,5,9,10-tetrone, a molecule studied intensively for application in organic batteries, and whose experimental crystal structure is reported by us in this study. We emphasize here that while CSP for the molecules studied here can also be achieved by other methods, DFTB3 will be more transferable to other redox-active organic materials when compared to force field-based methods and less computationally intensive when compared to DFT. Our results reveal CALYPSO/DFTB/DFT to be a promising approach for the CSP of a wide variety of organic molecules and lay the groundwork for future CSP studies of other organic molecules used for electrode materials in batteries.

## Methods

### Computational Methods

CSP was performed using the PSO algorithm as implemented in the CALYPSO software, in conjunction with the DFTB3 method implemented in DFTB+ (version 21.1).^15,33^ We performed structure predictions of selected molecules from the X23 data set and some quinones.^23,34,35^ The investigated molecules were grouped into three categories based on the nature of the dominant intermolecular interactions, namely, van der Waals (vdW) interactions, hydrogen bonding, and mixed bonding (Figure 1). Mixed bonding indicates the dominance of both vdW interactions and hydrogen bonding. The number of molecules per unit cell varied depending on the molecule of interest. Further details are provided in Table 1. The geometry of the investigated molecules was first optimized in the gas phase using the B3LYP^36^ functional and the 6–311++G** basis set.^37^ The optimized structures were then used as initial geometries for crystal structure prediction. The candidate structures were optimized in two steps using the DFTB3 method with the 3OB parameter set and dispersion corrections.^19,21^ In the first and second optimization steps, the tolerance for the maximum change in any charge between two consecutive self-consistent field iterations was set to 10^–5^ a.u. and 10^–6^ a.u., respectively. In both steps, optimization was considered completed when the force component with the maximal absolute value became less than 10^–4^ a.u. We utilized values of 0.746 au, 4.191 au, and 3.209 au for the *a*_1_, *a*_2_ and *s*_8_ parameters in the Becke-Johnson damping dispersion correction, respectively.^38,39^ The Hubbard derivative values used are listed in Table 2. The number of predicted structures per generation varied based on the molecule of interest, with details provided in Table 1. 50 generations were obtained for all molecules (default setting in CALYPSO) other than carbon dioxide, for which the CSP converged in the tenth generation. The number of structures per generation was chosen to be 5 or 10 based on the computational expense or the speed of convergence for the molecule under study. Details of some CSP convergence analysis are provided in section 1 of the Supporting Information (SI).

**Figure 1:**
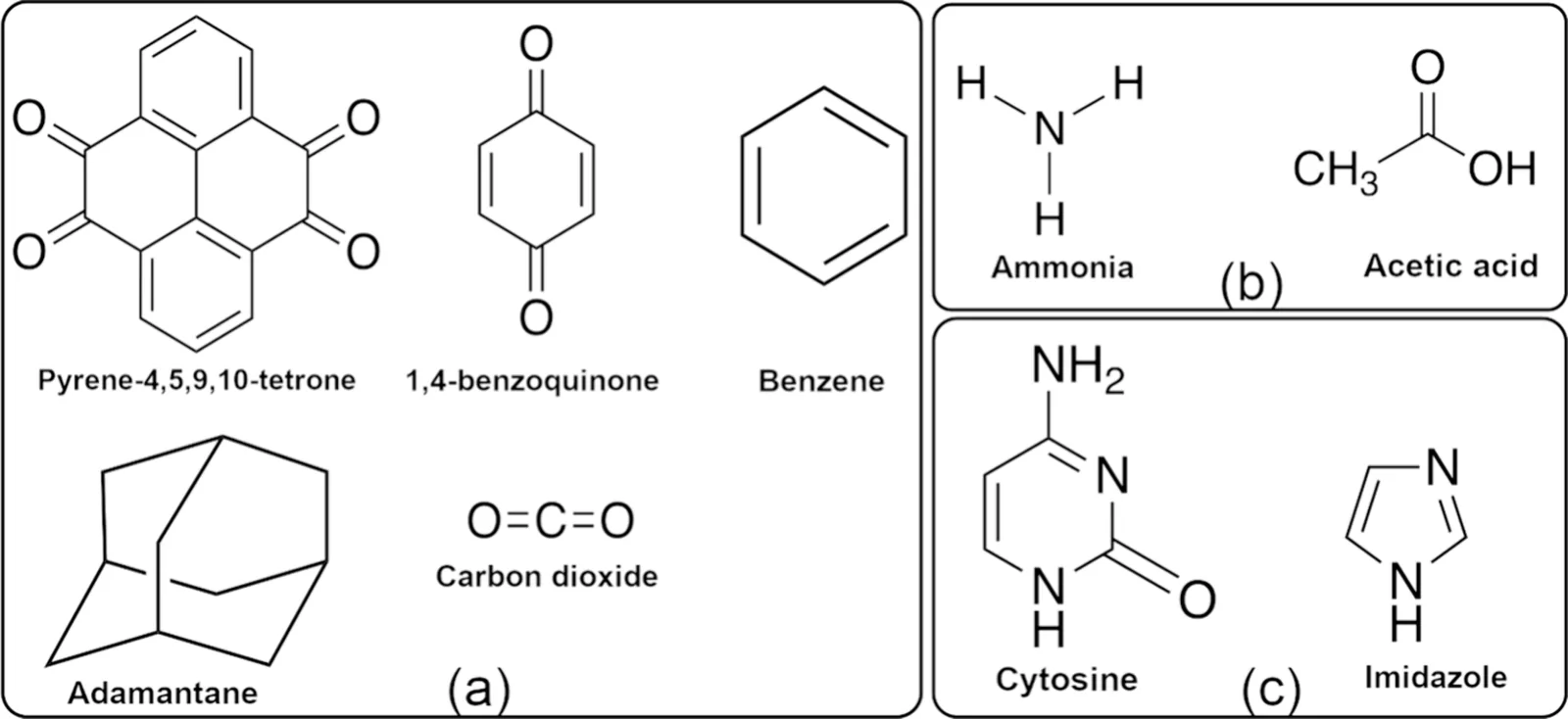
Pictorial representation of the molecules selected for crystal structure prediction, categorized based on the nature of the dominant intermolecular interactions. (a) van der Waals interactions, (b) hydrogen bonding, and (c) mixed bonding.

**Table 1:** Input Parameters for CSP Using CALYPSO/DFTB+

interaction type	molecule	minimum distance between molecules (Å)^a^	target unit cell volume (Å^3^)^b^	*Z* ^c^	structures/generation^d^
H-bonding	ammonia	1.50 (2.36)	125 (129)	4	5
	acetic acid	1.50 (1.83)	290 (297)	4	10
mixed bonding	cytosine	1.45 (2.00)	470 (472)	4	10
	imidazole	1.70 (1.86)	300 (349)	4	5
vdW	adamantane	1.95 (2.37)	370 (384)	2	10
	benzene	2.40 (2.54)	450 (474)	4	10
	carbon dioxide	3.00 (3.18)	185 (178)	4	10
	1,4-benzoquinone	2.00 (2.53)	250 (260)	2	5
	pyrene-4,5,9,10-tetrone	2.00 (2.34)	500 (521)	2	5

a Numbers in parentheses represent minimum distance between molecules in selected experimental structures.

b Numbers in parentheses represent unit cell volume in selected experimental structures.

c Number of molecules per unit cell.

d 50 generations were carried out for every molecule except CO_2_.

**Table 2:** DFTB3/3OB Hubbard Parameters in Atomic Units, Damping Exponent ζ = 4.00 ^21^

atom	*U* ^*d*^
H	–0.1857
C	–0.1492
O	–0.1575
N	–0.1535

50 of the lowest DFTB3-energy structures for each molecule were reoptimized using a generalized gradient approximation (GGA) functional, namely, Perdew–Burke–Ernzerhof (PBE),^40^ with D3 dispersion corrections. The core electrons were treated using the projector augmented wave (PAW) method.^41^ The PAW parameters were obtained from a standard solid state pseudopotential library (SSSP Efficiency).^42^ The default values for convergence threshold for total energy, forces, and dispersion corrections as implemented in the Quantum Espresso (QE) (version 6.4.1) software were used.^43^ All PBE calculations were performed using a 60 Ry plane-wave cutoff. The dense Monkhorst–Pack uniform *k*-grid for each molecule is specified in Table S2 of the SI.^44^ Henceforth, PBE and DFTB3 will be used to refer to PBE-D3/PAW, and DFTB3-D3/3OB methods, respectively. A comparison of DFTB3 and PBE rankings for low-energy polymorphs of all the molecules studied here as well as a comprehensive comparison of these rankings for some of the molecules is provided in the SI.

Our speed tests (Table S3) show that DFTB3 is inherently about 50–200 times faster than PBE. However, due to different optimization routines and optimization convergence criteria when using DFTB+ (for DFTB3 calculations) and Quantum Espresso (for PBE calculations), geometry/lattice optimization with DFTB3 is about 3–5 times faster than with PBE. This is a significant reduction in computational time since CSP with DFT alone can take up to a few weeks for a molecule like cytosine. Post the DFTB3 structure prediction, the additional step of PBE reoptimization takes less than or approximately an hour per candidate crystal structure for most molecules studied here, and about 4–5 h for some (Table S4). The reoptimization can be carried out simultaneously for several candidate crystal structures, making this step computationally nonprohibitive.

The CrystalCMP software was used to identify duplicate crystal structures by calculating the packing similarity (PS_*a,b*_) between two overlapping molecular clusters, using a threshold value of 0.1 set by Rohlíček et al.^45,46^ After overlapping the representative molecular clusters of the compared crystal structures such as to minimize the root-mean-square deviation between their atomic coordinates, the program identifies related molecular pairs between the two clusters based on the distance between molecular centers. The packing similarity PS_ab_ is calculated as PS_ab_ = D_c_ + X $\frac{A_{d}}{180}$ , with D_c_ being the average distance between the molecular centers of the related molecular pairs (in *Å*) and *A*_*d*_ being the average angle between them (in degrees). X is a user-defined weight that affects the contribution of *A*_*d*_ to PS_ab_ (default value = 100). Further details can be found in ref. 45,46 The overall workflow for CSP used in this study is summarized in Figure 2.

**Figure 2:**
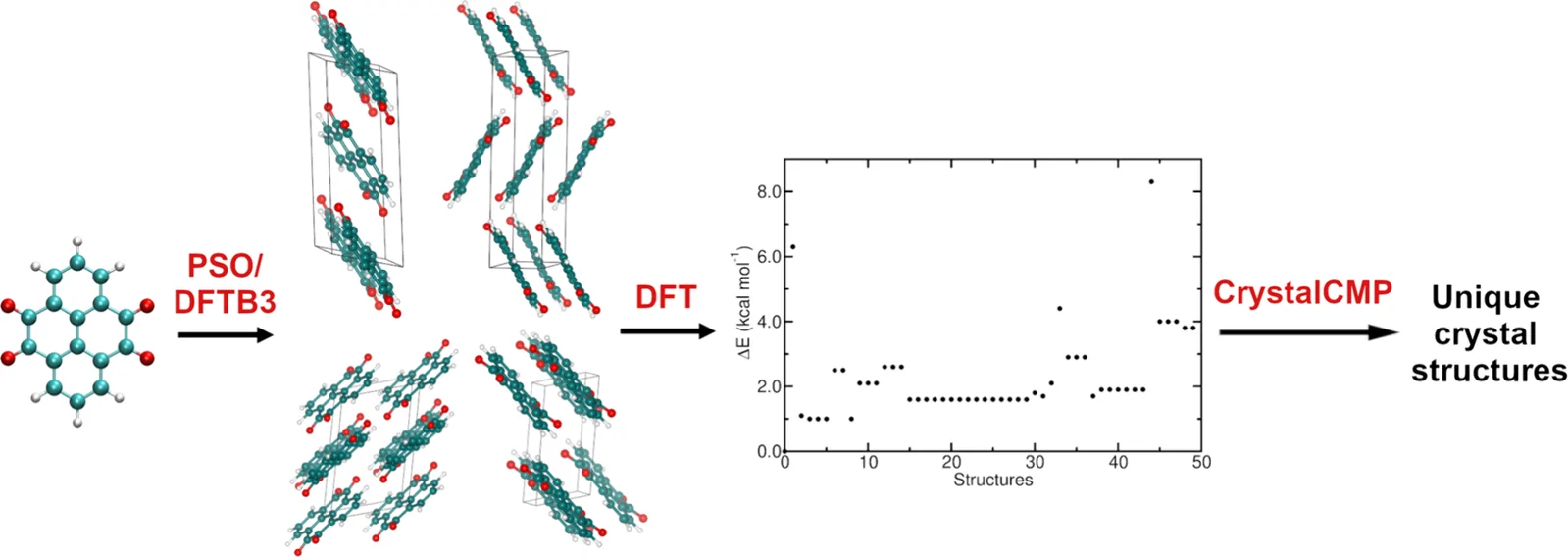
CSP workflow adopted in this study: After the generation of candidate crystal structures using PSO in conjunction with DFTB3-D3/3OB, the 50 lowest energy structures are reoptimized with DFT (PBE-D3/PAW) and reranked. This is followed by the identification of unique crystal structures using the CrystalCMP software.

The lattice energy (U_latt_) corresponding to the most stable crystal structure of each molecule was calculated using eq 2, where U_s_ is the total energy of the unit cell, Z is the number of molecules per unit cell, and U_g_ is the total energy of a molecule in the gas phase. The gas phase calculations were carried out in Quantum Espresso (version 6.4.1) using the PBE functional and additional parameters as described earlier. We used a value of 25 Å for *a*, *b*, and *c* in these calculations.

$$U_{\mathrm{latt}}=\frac{U_{s}}{Z}-U_{g}$$ 2

### Pyrene-4,5,9,10-tetrone (PTO) Crystal Synthesis

The PTO single crystal was grown via vaporization method. Specifically, to a 5 mL glass vial, 1 mg PTO and 2 mL dichloromethane (DCM) were added to afford a light-yellow PTO solution. The vial was sealed with a plastic cap that was poked by a needle (0.5 mm × 22 mm), then transferred to a 4 °C refrigerator to slow down the vaporization of DCM. Needle-like single crystals appeared on the bottom of the solution after around 10 days of sitting in the refrigerator. The crystals were kept in the remaining solvent and used for single-crystal X-ray diffraction measurement.

## Results and Discussion

### Hydrogen Bonding Class

#### Ammonia

Solid ammonia is known to have different phases with increasing pressure. In the literature, several phases have been identified with distinctive physical properties and space groups.^47^ All the experimentally reported phases were obtained for deuterated NH_3_. Phase I has ordered molecules packed in a cubic lattice (*P*2_1_3 space group) at 0.00 GPa,^48^ phase II has disordered molecules packed in a hexagonal lattice (*P*6_3_/*mmc* space group) at 0.51 GPa,^49^ phase III has disordered molecules packed in a face-centered lattice (*Fm*3*m* space group) at 1.28 GPa,^50^ and phase IV has ordered molecules packed in an orthorhombic lattice (*P*2_1_2_1_2_1_ space group) at 9.00 GPa.^51^ In addition, phase V found at ∼14.00 GPa is speculated to belong to the *P*2_1_2_1_2_1_ space group.^52,53^ Figure 3 shows the available experimental crystal structures of solid NH_3_ that contain ordered molecules, viz., phases I and IV.^48,51^

**Figure 3:**
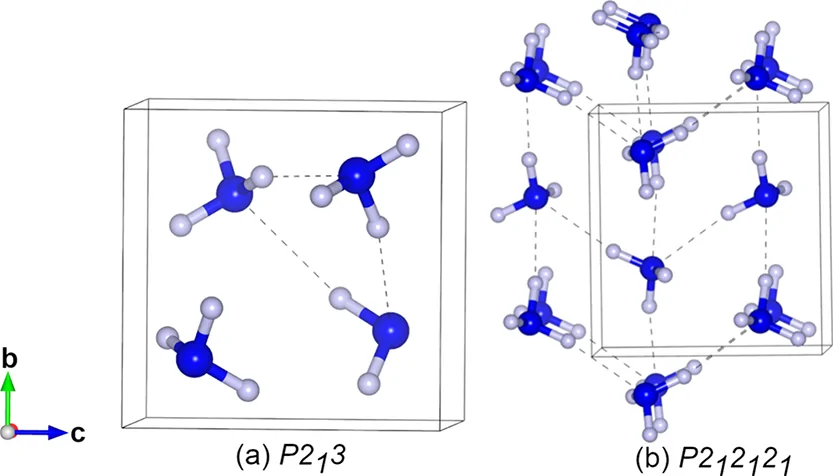
Experimental crystal structures of solid NH_3_. (a) Phase I (*P*2_1_3) and (b) phase IV (*P*2_1_2_1_2_1_). Dark blue and gray spheres represent N and D atoms, respectively.

We performed CSP for NH_3_ using CALYPSO/DFTB+ and reoptimized 50 of the most favorable DFTB3 structures using PBE. We calculated PS_*a,b*_ to identify duplicate structures that yielded 6 polymorphs in different space groups (Figure 4, Tables S5 and S6). The most stable polymorph identified computationally matches the experimental phase I at 0.00 GPa, belonging to the *P*2_1_3 space group (Table S6, Figures 3a and 4a).^52^ The calculated PS_*a,b*_ values of the 7 crystal structures belonging to this space group are in the 0.01–0.02 range and within the accepted tolerance of PS_*a,b*_ = 0.1 (Figure S1). These structures have three ammonia molecules donating and accepting a hydrogen bond forming a triangular pattern (Figures 3a and 4a).

**Figure 4:**
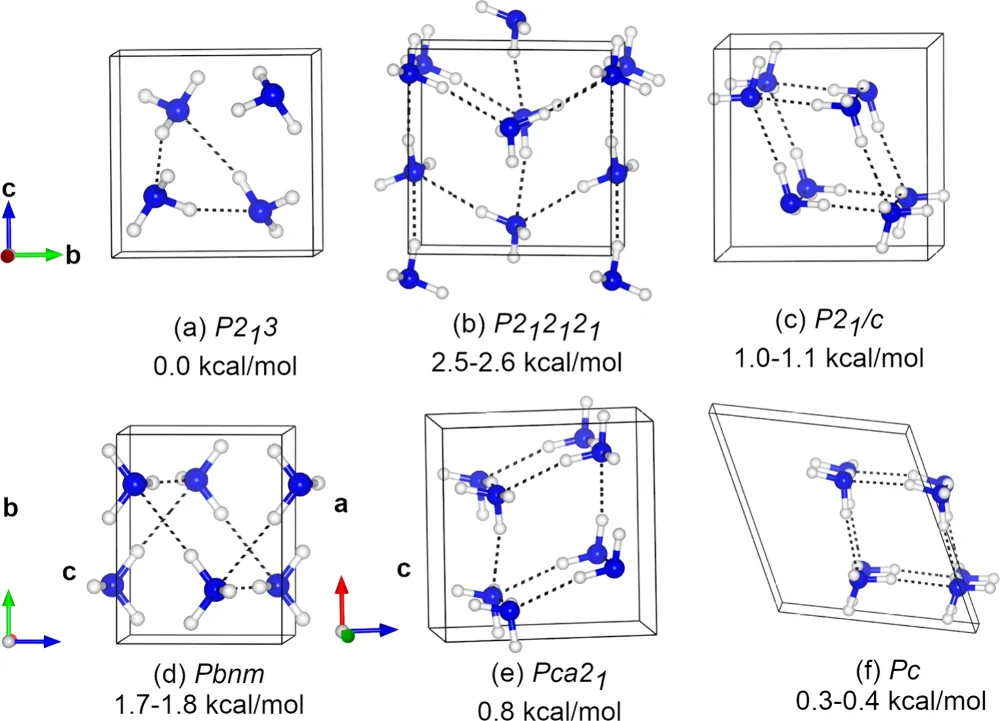
Predicted crystal structures of solid NH_3_. (a) *P*2_1_3 (phase I), (b) *P*2_1_2_1_2_1_ (phase IV), (c) *P*2_1_/*c*, (d) *Pbnm,* (e) *Pca*2_1_, and (f) Pc space groups. Dark blue and white spheres represent N and H atoms, respectively. The PBE energy relative to the most stable crystal structure is shown below each crystal structure.

The additional polymorphs are predicted to be within ∼2.5 kcal mol^–1^ of the most stable polymorph (Tables S5 and S6). These include the experimentally observed phase IV at 9.00 GPa with the *P*2_1_2_1_2_1_ space group (Figure 4b), polymorphs with the *Pbnm* and *Pca*2_1_ space groups in the orthorhombic crystal system (Figure 4d,e), and polymorphs with the *P*2_1_/*c* and Pc space groups in the monoclinic crystal system (Figure 4c,f). The polymorph with the *P*2_1_/*c* space group was first predicted by Pickard et al. using the PBE/USSP method^52^ (Figure 4c).

Overall, our methodology identifies the experimentally observed phase I at 0.0 GPa to be the most stable polymorph of NH_3_. Phase IV observed at 9.00 GPa, the only other experimentally observed phase with ordered molecules, and the *P*2_1_/*c* polymorph identified by a previous computational study were also found to be low energy polymorphs.

#### Acetic Acid

There are two experimentally observed polymorphs of acetic acid. One of the polymorphs has an orthorhombic crystal structure (space group *Pna*2_1_) which can exist at low and high temperatures, 40 and 278 K, respectively (Figure 5a). The other polymorph has a monoclinic structure (space group *P*2_1_/*n*) at 289 K (Figure 5b). Both polymorphs have four molecules per unit cell.^54–56^

**Figure 5:**
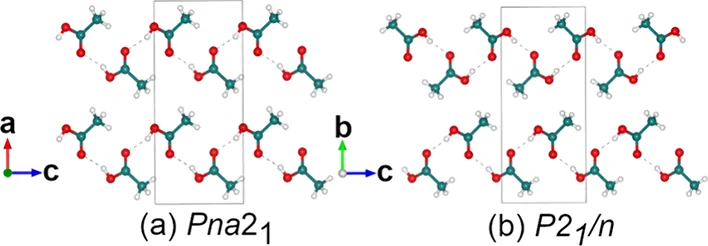
Experimental crystal structures of acetic acid. (a) Orthorhombic (*Pna*2_1_) and (b) monoclinic (*P*2_1_/*n*). White, cyan, and red spheres represent H, C, and O atoms, respectively.

Following CSP for acetic acid using CALYPSO/DFTB+, we reoptimized 50 of the most favorable DFTB3 structures using PBE. We calculated PS_*a,b*_ to identify duplicate structures that yielded four polymorphs in orthorhombic and monoclinic space groups (Figure 6, Tables S7 and S8). The most stable polymorph from PBE has lattice parameters and packing similar to the experimental monoclinic structure (Figures 5b and 6b and Table S8). The PS_*a,b*_ value of 0.13 between the PBE and experimental monoclinic structures indicates that they are identical. The experimental and predicted orthorhombic crystal structures do not agree with each other. Additional lattice parameters are provided in Table S8.

**Figure 6:**
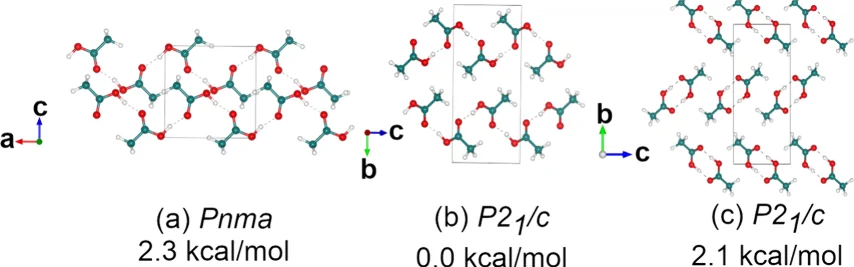
Predicted crystal structures of acetic acid. (a) Orthorhombic (*Pnma*), (b) monoclinic structure 20 (*P*2_1_/*c*), and (c) monoclinic structure 43 (*P*2_1_/*c*). The PBE energy relative to the most stable crystal structure is shown below each crystal structure.

### Mixed Bonding Class

#### Cytosine

Anhydrous cytosine has an orthorhombic crystal structure (*P*2_1_2_1_2_1_ space group) with four molecules per unit cell. The hydrogen bonding and π-stacking interactions between the cytosine molecules are illustrated in Figure 7a,b, respectively. Each cytosine molecule is involved in six hydrogen bonds that arise from the O atom accepting two hydrogen bonds and the N atoms having a dual function of accepting and donating hydrogen bonds.

**Figure 7:**
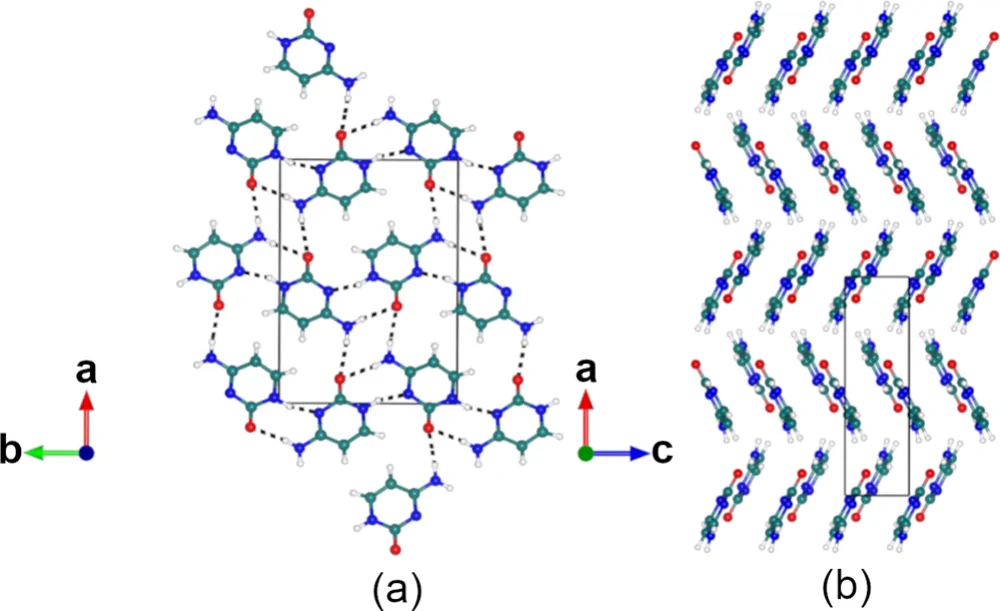
Experimental crystal structure of cytosine. (a) Front view showing the hydrogen bonds. (b) Side view showing the π–stacking interactions. White, cyan, dark blue, and red spheres represent H, C, N, and O atoms, respectively.

We computationally predict an orthorhombic structure (*P*2_1_2_1_2_1_ space group) similar to the experimental structure to be the most stable polymorph (Figure 8). The optimized lattice parameters of the orthorhombic structure are reported in Table S9. The PS_*a,b*_ value between the experimental and PBE structures was found to be only 0.124. Other predicted polymorphs were higher in energy by 10–41 kcal/mol.

**Figure 8:**
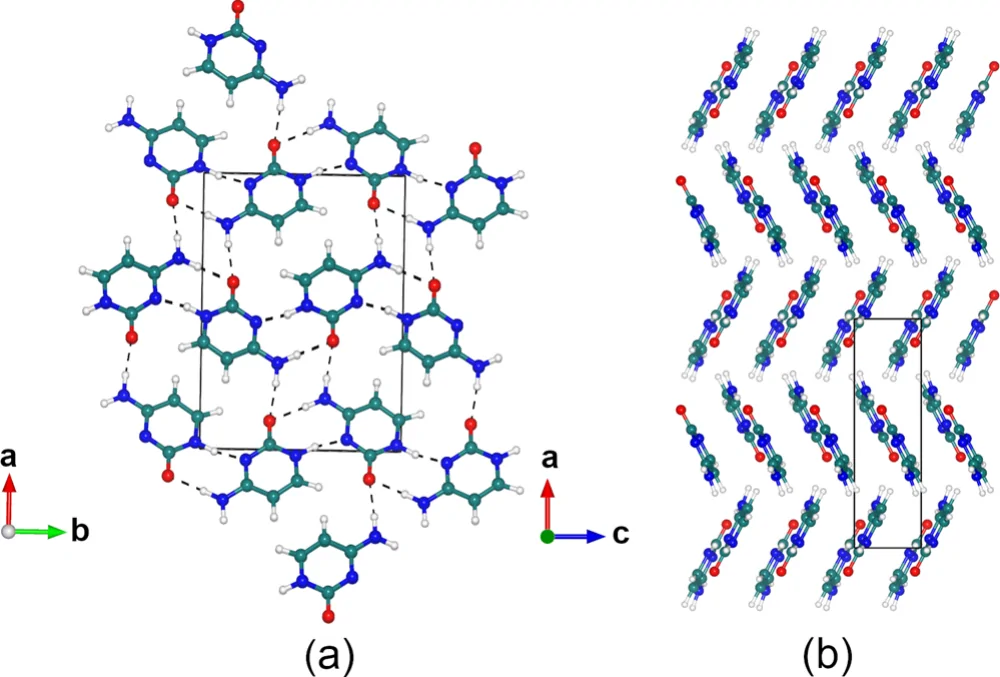
Predicted crystal structure of cytosine. (a, b) Front and side views, respectively.

#### Imidazole

Many experimental crystal structures of imidazole belong to the monoclinic and orthorhombic crystal lattices with four and eight molecules per unit cell, respectively, and have similar lattice parameters. We utilized CrystalCMP to group similar experimental structures and calculated the mean of the lattice parameters for structures that were similar (Tables S10 and S11). Representative crystal structures with monoclinic and orthorhombic crystal lattices are shown in Figure 9. The mixed bonding arises from the hydrogen bonds present between the nitrogen atoms and π–stacking interactions.

**Figure 9:**
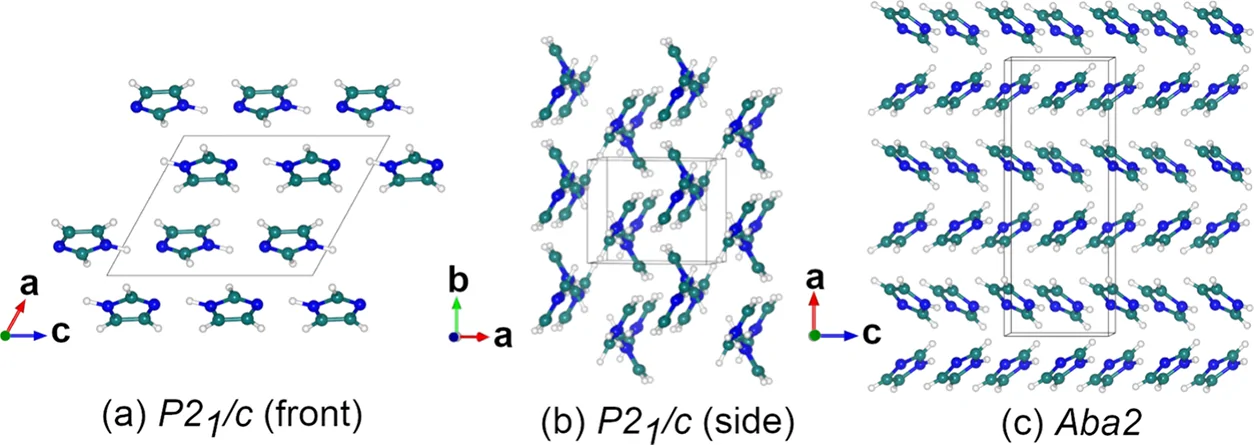
Experimental crystal structures of imidazole. (a, b) Front and side views, respectively, of the monoclinic crystal lattice (CSD code IMAZOL01). (c) Orthorhombic crystal lattice (CSD code IMAZOL26). White, cyan, and dark blue spheres represent H, C, and N atoms, respectively.

Our calculations set up to allow a maximum of four molecules per unit cell predicted the monoclinic structure to be the most stable. The most stable polymorph from PBE (Figure 10a,b) had packing and lattice parameters similar to group 1 of experimental monoclinic structures, PS_*a,b*_ = 0.17 (Figure 9a,b, Tables S12 and S13). Details of other computationally identified polymorphs are provided in Figure 10c,d and Tables S12 and S13.

**Figure 10:**
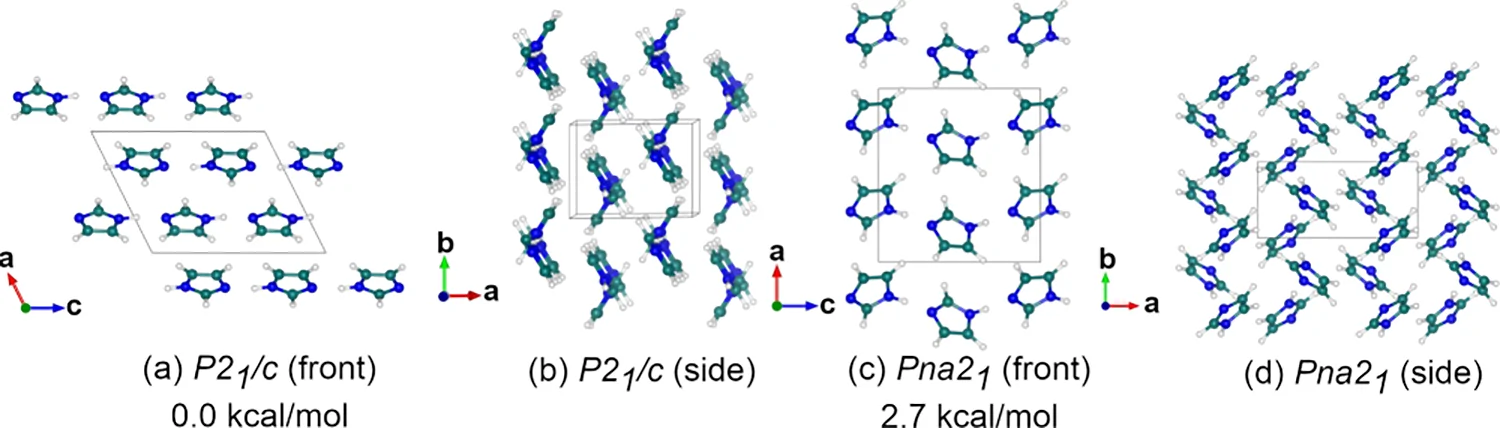
Predicted crystal structures of imidazole. (a, b) Front and side views of structure 14 (*P*2_1_/*c*), respectively. (c, d) Front and side views of structure 1 (*Pna*2_1_), respectively. The PBE energy relative to the most stable crystal structure is shown below each crystal structure.

### vdW Class

Results for carbon dioxide and benzoquinone are presented in Section 5 of the SI.

#### Adamantane

Experimentally, adamantane exists in two phases depending on temperature. At room temperature (Figure 11a), adamantane has a face-centered cubic crystal structure (space group *Fm*3*m*) with four molecules in the unit cell and *a* = 9.450 Å (CSD code ADAMAN).^57^ At 163 and 188 K (Figure 11b), the structure exists as a tetragonal crystal lattice (space group *P*4̅2_1_ *c*) with two molecules in the unit cell. At 163 K, *a* = 6.600 Å, *c* = 8.810 Å (CSD code ADAMAN02), and at 188 K, *a* = 6.639 Å, *c* = 8.918 Å (CSD code ADAMAN08).^58^

**Figure 11:**
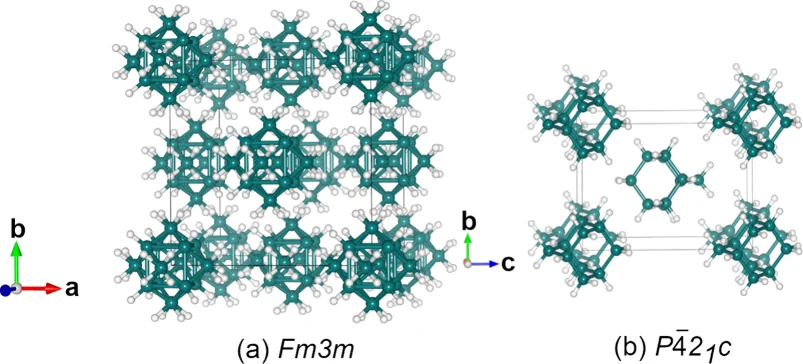
Experimental crystal structures of adamantane. (a) Face-centered cubic structure (*Fm*3*m*) at room temperature, CSD code ADAMAN. (b) Tetragonal structure (*P*4̅2_1_ *c*) at 188 K, CSD code ADAMAN08. White and cyan spheres represent H and C atoms, respectively.

Keeping in mind the computational cost of CSP for a large molecule like adamantane, we sampled adamantane crystal structures with two molecules per unit cell. All the 50 lowest energy polymorphs from DFTB3 were found to belong to the *P*4̅2_1_ *c* space group and were identical, PS_*a,b*_ = 0.01. Figure 12 shows one of the predicted crystal structures (structure 1) optimized using PBE, *a* = 6.554 Å, and *c* = 8.867 Å. The lattice parameters are similar to those for the experimental tetragonal crystal structures.

**Figure 12:**
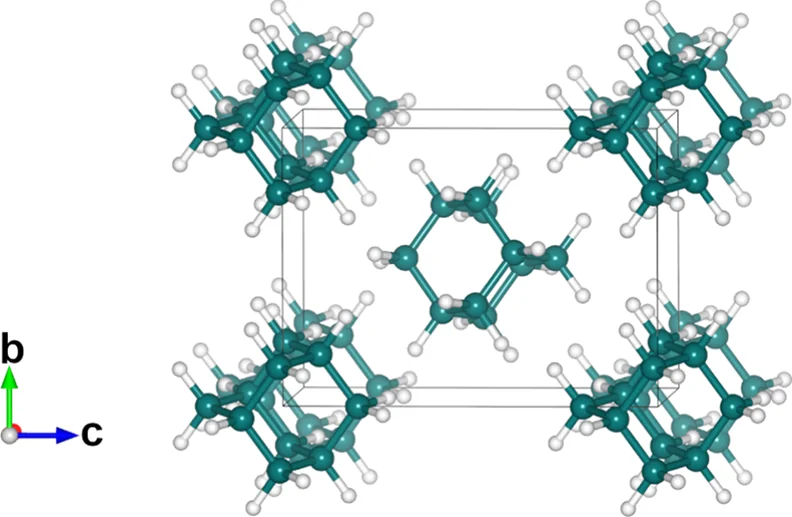
Predicted crystal structure of adamantane, structure 1 (*P*4̅2_1_ *c*).

#### Benzene

Experimentally, orthorhombic (*Pbca* space group) and monoclinic (*P*2_1_/*c* space group) crystal structures for benzene have been reported (Figure 13). The orthorhombic and monoclinic structures have four and two molecules per unit cell, respectively. Some of the experimental structures from the Cambridge Crystallographic Data Center (CCDC) had similar packing with slightly different lattice parameters.^59^ We utilized CrystalCMP to group similar experimental structures and calculated the mean of the lattice parameters for structures that were similar. These lattice parameters are listed in Tables S14 and S15.

**Figure 13:**
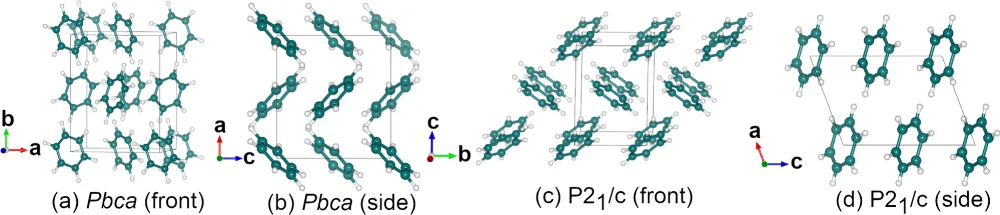
Experimental crystal structures of benzene. (a, b) Front and side views of the orthorhombic structure (*Pbca*), respectively, CSD code BENZEN. (c, d) Front and side views of the monoclinic structure (*P*2_1_/*c*), respectively, CSD code BENZEN16. White and cyan spheres represent H and C atoms, respectively.

We predicted structures that were grouped into three polymorphs, orthorhombic (*Pbca*), tetragonal (*P*4_3_2_1_2), and monoclinic (*P*2_1_/*c*). Similar to experiments, there are four and two molecules per unit cell for the orthorhombic and monoclinic structures, respectively. We rank the orthorhombic structure as the most stable, followed by the monoclinic structure (Table S16). The predicted orthorhombic structure (Figures 14a, b) has the same packing as the experimental structure (Figure 13a,b). Similarly, the experimental monoclinic structure (Figure 13c,d) has the same packing as the predicted monoclinic structure (Figure 14c,d) with a PS_*a,b*_ of 0.1. Figure 14e shows the tetragonal structure, which is first reported here. The lattice parameters for the predicted structures are listed in Table S17.

**Figure 14:**
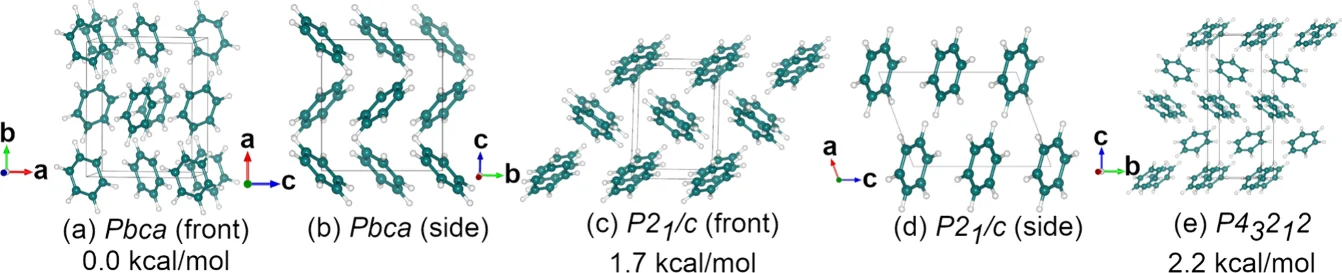
Predicted crystal structures of benzene. (a) and (b) Front and side views of orthorhombic structure 25 (*Pbca*), respectively. (c) and (d) Front and side views of monoclinic structure 1 (*P*2_1_/*c*), respectively. (e) Tetragonal structure 4 (*P*4_3_2_1_2). The PBE energy relative to the most stable crystal structure is shown below each crystal structure.

### Test Case: Pyrene-4,5,9,10-tetrone (PTO)

PTO is a quinone with four redox-active carbonyl groups that has been explored a lot for organic electrodes in batteries due to its high charge capacity and ability to store a wide variety of cations.^9,60–68^ The experimental crystal structure of PTO was obtained by us (Table 3, Tables S22–S25, and Figure 15) and the ability to computationally predict its crystal structure is of importance for our future research involving this molecule. The experimental crystal structure has two molecules in each unit cell and belongs to the monoclinic crystal system (*P*2_1_/*c* space group). The experimental lattice parameters are *a* = 3.728 Å, *b* = 15.405 Å, *c* = 9.243 Å, β = 100.45°, V = 522.08 Å^3^. Figure 15b depicts the π-stacking interactions between the PTO molecules. The experimentally observed structure of PTO (CCDC number 2250982) has the classical herringbone packing.

**Figure 15:**
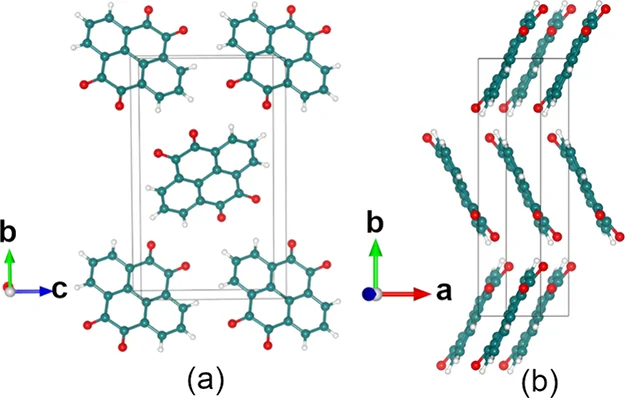
Experimental crystal structure of PTO. (a) Front view of the unit cell; (b) side view showing π–stacking interactions in the unit cell. White, cyan, and red spheres represent H, C, and O atoms, respectively.

**Table 3:** Crystal Data and Structure Refinement for PTO

identification code	PTO	
empirical formula	C_16_H_6_O_4_	
formula weight	262.21	
temperature	123(2) K	
wavelength	1.54178 Å	
crystal system	monoclinic	
space group	*P*2_1_/*c*	
unit cell dimensions	*a* = 3.7283(2) Å	α = 90°
	*b* = 15.4053(8) Å	β = 100.452(4)°
	*c* = 9.2432(5) Å	γ = 90°
volume	522.08(5) Å^3^	
Z	2	
density (calculated)	1.668 Mg/m^3^	
absorption coefficient	1.018 mm^–1^	
F(000)	268	
crystal size	0.30 × 0.05 × 0.04 mm^3^	
theta range for data collection	5.744 to 66.428°	
index ranges	–4 ≤ *h* ≤ 4, 0 ≤ *k* ≤ 18, 0 ≤ *l* ≤ 10	
reflections collected	6505	
independent reflections	902 [R(int) = 0.1063]	
completeness to theta = 66.428°	97.6%	
absorption correction	empirical	
max. and min transmission	0.7518 and 0.2612	
refinement method	full-matrix least-squares on F^2^	
data/restraints/parameters	902/0/92	
goodness-of-fit on F^2^	1.078	
final R indices [I > 2sigma(I)]	R1 = 0.0959, wR2 = 0.2394	
R indices (all data)	R1 = 0.1014, wR2 = 0.2495	
largest diff. peak and hole	0.476 and −0.353 e Å^–3^	

Ranking the predicted crystal structures in terms of energy, 38 of the most stable structures belonged to the monoclinic crystal system with the *P*2_1_/*c* space group. PS_*a,b*_ calculations revealed most of these crystal structures to be duplicates, leading to a total of four different monoclinic polymorphs (Table S20, Figure 16). Computations predict the experimental structure to be the most stable polymorph. Figure 16a,b and Table 4 show that this polymorph has the same packing style and similar lattice parameters as the experimental crystal structure (Figure 15). Lattice parameters for additional polymorphs are provided in Table S21.

**Figure 16:**
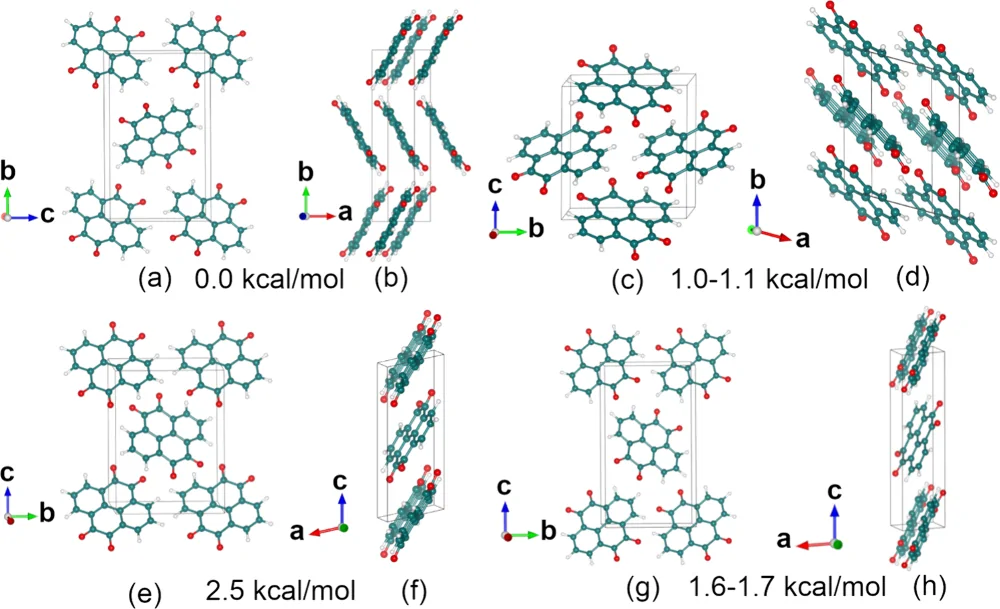
Predicted PTO crystal structures. (a, b), crystal structure 1, (c, d) crystal structure 3, (e, f) crystal structure 7, and (g, h) crystal structure 16. (a, c, e, g) Front view of the respective crystal structures. (b, d, f, h) Side view of the respective crystal structures. The PBE energy relative to the most stable crystal structure is shown below each crystal structure.

**Table 4:** Optimized Lattice Parameters for the Predicted Crystal Structure of PTO in the *P*21/*c* Space Group

crystal structure	lattice parameter	expt.	PBE
1	*a* (Å)	3.728	3.781
	*b* (Å)	15.405	15.271
	*c* (Å)	9.243	9.175
	β (°)	100.45	98.87
	*V* (Å^3^)	522.08	523.45

### Lattice Energy

Crystal lattice energy plays a crucial role in rationalizing the stability of predicted structures. It is the energy released when molecules interact to form a crystal. Molecular crystals are often held together mainly by intermolecular forces such as dispersion forces. Therefore, dispersion corrections are paramount for molecular crystal structure prediction and calculation of lattice energy. Lattice energies calculated using PBE-D3 are listed in Table 5 and compared to experimental values. The lattice energies of adamantane, CO_2_, and cytosine are underestimated by 3.6, 4.5, and 6.4 kJ/mol, respectively, compared to experiments. For acetic acid, ammonia, and imidazole, the lattice energy is overestimated by 0.1, 5.4, and 3.0 kJ/mol, respectively. For benzene, our predicted orthorhombic and tetragonal polymorphs have four molecules per unit cell, while the monoclinic polymorph has two molecules per unit cell. Therefore, we calculated the lattice energy for each polymorph to accurately rank these three polymorphs. The orthorhombic polymorph is found to be the most stable, followed by the monoclinic and tetragonal polymorphs. Overall, PBE-D3 performs relatively well in describing the lattice energies of molecular organic crystals, with a mean absolute error of only 4.3 kJ/mol or ∼1 kcal/mol and the maximum error being only 6.4 kJ/mol or ∼1.5 kcal/mol.

**Table 5:** Calculated Lattice Energies in kJ/mol for Predicted Crystal Structures

interaction type	molecule	structure^a^	space group	U_latt._ (exp)^b^	U_latt._ (calc)^c^
H-bonding	ammonia	cubic	*P*2_1_3	–37.2	–42.6 (−5.4)
	acetic acid	monoclinic	*P*2_1_/*c*	–72.8	–72.9 (−0.1)
mixed bonding	cytosine	orthorhombic	*P*2_1_2_1_2_1_	–162.8	–156.4 (+6.4)
	imidazole	monoclinic	*P*2_1_/*c*	–86.8	–89.8 (−3.0)
vdW	adamantane	tetragonal	*P*4̅2_1_ *c*	–69.4	–65.8 (+3.6)
	benzene	orthorhombic	*Pbca*	–55.3	–51.5 (+3.8)
		monoclinic	*P*2_1_/*c*		–49.7 (+5.6)
		tetragonal	*P*4_3_2_1_2		–49.2 (+6.1)
	CO_2_	cubic	*Pa*3̅	–28.4	–23.9 (+4.5)
	BQ	triclinic	*P*1̅		–73.4
	PTO	monoclinic	*P*2_1_/*c*		–139.0

a The most stable structure from PBE.

b Reference 23, benzene and cytosine use revised values from ref 69.

c U_latt_ calculated using PBE-D3. Numbers in parentheses are errors compared to experiments.

### CSP in the Absence of Experimental Volume Data

Based on our studies, we propose that in the absence of experimental unit cell volume data, the target volume for CSP can be approximated from the dimensions and overall shape of the molecule, the type of intermolecular interactions, and the number of molecules in the unit cell. The detailed procedure is described in Section 9 of the SI. Table 6 outlines the calculated unit cell volumes for the various molecules studied here (also see Table S26). The volume of most of the molecules can be calculated by assuming a cylindrical molecular shape, other than for adamantane and acetic acid, which were assumed to have spherical and rectangular shapes, respectively. We extended the radius of the cylinder/sphere or a dimension of the rectangle by a value between 0.75–1.00 Å depending on the type of intermolecular interactions and the empty space in the geometrical shape enclosing the molecule (further details have been provided in the SI). The unit cell volumes calculated using this procedure are found to be close to the experimental and target unit cell volumes for the molecules of interest (Table 6). Hence, we speculate that in the future, using the calculated volume as target volume in a CSP study, it will be possible to predict crystal structures based solely on the identity of the molecule, the nature of intermolecular interactions, and the number of molecules in the unit cell. We note here that since DFTB3-D3 underestimates unit cell volumes by about 30–40 *Å* ^3^ for most of the molecules studied here, and by a larger extent for molecules with planar aromatic ring structures, the target unit cell volume in the workflow should be set to be ∼30 *Å* ^3^ lower than the experimental or calculated unit cell volume so that DFTB can successfully locate the experimental crystal structure (also see Section 10 in the SI).

**Table 6:** Calculated Unit Cell Volumes for the Molecules of Interest

interaction type	molecule	expt. volume (Å^3^)	target volume (Å^3^)^a^	average volume (Å^3^)^b^	calc. volume (Å^3^)^c^	Z^d^
H-bonding	ammonia	129	125	88	123	4
	acetic acid	297	290	260	294	4
mixed bonding	cytosine	472	470	441	451	4
	imidazole	349	300	309	335	4
vdW	adamantane	384	370	349	360	2
	benzene	474	450	404	438	4
	CO_2_	178	185	160	157	4
	BQ	260	250	218	262	2
	PTO	521	500	480	513	2

a User-defined target volume used in the CSP calculations in this study.

b Average of the DFTB3 volumes of the 50 most favorable structures predicted by DFTB3.

c Volume calculated as outlined above.

d Number of molecules per unit cell.

## Conclusions

In this study, we have shown that the DFTB3-D3 method in conjunction with the particle swarm optimization algorithm implemented in CALYPSO, is an efficient approach for the determination of the low energy polymorphs of organic molecules with different kinds of intermolecular interactions, ranging from vdW to hydrogen-bonding to a combination of both. DFT-D3 calculations on the lowest ranked polymorphs from DFTB3-D3 leads to their proper ranking and identification of the most stable structure, which is found to be in agreement with the experimental crystal structure. This method correctly predicts the crystal structure of pyrene-4,5,9,10-tetrone, a molecule studied intensively for application in organic batteries, whose experimental crystal structure is also reported in this study. For all the molecules studied here, the experimentally observed structure consistently appears within the 35 lowest energy structures generated by PSO/DFTB3, demonstrating the reliability of this approach for the kinds of molecules studied here.

To the best of our knowledge, this is not only the first application of CALYPSO to organic molecules, but also the first CSP study involving the systematic application of the DFTB3 method to different classes of organic molecules, including quinones which are relevant to organic electrode materials for rechargeable batteries.

The calculated lattice energies using PBE-D3 were found to have a mean absolute error of only 4.3 kJ/mol or ∼1 kcal/mol, the maximum error being only 6.4 kJ/mol or ∼1.5 kcal/mol. The trends observed in the calculated lattice energies for different molecules are similar to those observed by Reilly and Tkatchenko^23^ using the Tkatchenko-Scheffler (TS)^34^ and many-body dispersion (MBD) corrections with the PBE and hybrid PBE functionals.

We propose extensions of this CSP methodology to CSP problems where only the molecule of interest and the number of molecules in the unit cell are known. We show that the shape of the molecule, the nature of intermolecular interactions, and the number of molecules in the unit cell can be used to estimate the volume of the unit cell. This volume can be used as the target volume in CSP calculations in the absence of experimental volume data. Most organic CSP studies use a value of 1 for the number of asymmetric units (Z′) to limit the number of space groups to be sampled.^70^ This is mainly because most organic molecules tend to crystallize in the monoclinic, triclinic, and orthorhombic space groups. However, structures with Z′ > 1 may be overlooked upon using this approach. The PSO algorithm as implemented in CALYPSO tends to break the asymmetric unit, allowing us to sample more space groups.^71^

There exist fully unconstrained CSP methods^72,73^ for which a predefined target unit cell volume is not a required parameter and which hence provide greater flexibility. However, these methods can be especially useful in cases like high pressure discovery of novel polymorphs where volume reduces drastically, for modeling empty space in porous material like metal–organic frameworks without any bias or for identification of elusive metastable structures. They can result in a much higher computational cost due to the massive search space available and a higher risk of getting trapped in local minima.

The applicability of the current methodology to crystals with charged organic molecules remains to be investigated. While the geometry of different kinds of organic molecules in different redox states is described very well by DFTB3/3OB,^21,74^ the errors in the description of noncovalent interactions between charged organic molecules in the crystalline phase^20,25,26,32^ are not well-understood, and need to be investigated before application of the 3OB parameter set to crystals with charged organic molecules. Recent studies have carried out reparameterization of DFTB3 through machine-learning or added improved dispersion corrections for specific applications such as improving the description of π-stacking for sulfur-containing moelcules^75^ or studying large organic molecules and noncovalent interactions between them.^76^ Similar approaches can be adopted for further development of DFTB3 if required for the study of crystals with organic molecules in different redox states. For more complicated systems, an increase in the size of the candidate structure pool from DFTB may also be required since the global minimum may lie outside the 50 lowest energy structures from DFTB.

While the hybrid DFTB/DFT approach proposed here is much more efficient than using DFT alone and provides much more accuracy than traditional force fields, it faces competition from machine learning methods for CSP. Machine-learning methods are becoming increasingly popular for CSP with their much higher efficiency than traditional DFT and their high accuracy.^77–79^ They have also found reasonable success in the seventh CSP blind test.^80^ While they are very efficient at structure generation and ranking, the cost of training the machine learning potential can be extremely high for unconventional molecules.^80^ The transferability of the trained potential to molecules outside the training data set is also always a matter of concern. The Δ-ML model developed by Wengert et al.^27,28^ includes ML-based corrections to DFTB+TS and is found to be capable of describing lattice energies and energy ranking of organic crystals with comparable accuracy to PBE+MBD. However, in their study, the ML needed to be carried out for every new molecule of interest. Despite the fact that both DFTB3 and machine-learning methods suffer from concerns regarding transferability and the cost of reparametrizing/retraining, we have found DFTB3-D3 with the general purpose 3OB parameter set to work very well for CSP of the molecules of interest without any adjustment to the parameters. Hence, the methodology proposed here remains competitive even in the landscape of emerging machine-learning methods for CSP.

Overall, we show that CALYPSO/DFTB3-D3 with the existing 3OB parameter set, followed by postprocessing with DFT-D3, is an accurate and computationally efficient method for crystal structure prediction of the molecules investigated here and possibly other redox-active organic materials for which neither empirical force fields nor DFT may be an ideal choice.

## Supplementary Material





## Notes

Additional details regarding the computational methodology, and additional computational and experimental results (PDF)
Experimental crystal structure of pyrene-4,5,9,10-tetrone (CIF)

## ⊥

Pacific Northwest National Laboratory, Richland, WA, 99354, United States

## †

National University of Singapore, Singapore, 117575, Singapore

## #

Illinois State University, Normal, IL, 61761, United States
