# Supplementary material for: Efficient Organic Molecular Crystal Structure Prediction Using the Density Functional Tight-Binding Method
Source: ACS Phys Chem Au. 2026 Jun 20;6(5):1050–61. doi: 10.1021/acsphyschemau.6c00036 (PMC13614049; doi:10.1021/acsphyschemau.6c00036)
Supplement: Supplementary file 1 [file pg6c00036_si_001.pdf]

SUPPORTING INFORMATION

EFFICIENT ORGANIC MOLECULAR CRYSTAL STRUCTURE PREDICTION USING THE DENSITY FUNCTIONAL TIGHT-BINDING METHOD

Maureen M. Kitheka [a, ⊥], Yan Jing[b, †], Yan Yao[b], Puja Goyal [a, c, #, *]

[a] Department of Chemistry, State University of New York at Binghamton, Binghamton, NY 13902, USA

[b] Department of Electrical and Computer Engineering and Texas Center for Superconductivity (TcSUH), University of Houston, Houston, Texas 77204, USA

[c] Department of Chemistry, Illinois State University, Normal, IL 61761, USA

[⊥] Current address: Pacific Northwest National Laboratory, Richland, WA 99354, USA

[†] Current address: National University of Singapore, Singapore, 117575, Singapore

[#] Current address: Illinois State University, Normal, IL 61761, USA

[*] Corresponding author; Email: pgoyal@ilstu.edu

TABLE OF CONTENTS

Page

1. Crystal structure prediction (CSP) sampling convergence……………………………….S2
2. *k*-grids for each molecule………………………………………………….……......S3
3. Speed tests……………………………………………………………………..…... S3
4. Finding identical crystal structures……………………………………………………….S5
5. CSP results for carbon dioxide and benzoquinone……………………………………….S6
6. Relative energies and lattice parameters for various polymorphs of the studied molecules………………………………..……………………………………….S8
7. Experimental crystal structure of pyrene-4,5,9,10-tetrone (PTO)………………….…..S15
8. Average unit cell volume of DFTB3-predicted structures………………………………S17
9. Calculation of target volume in the absence of experimental crystal structure……......S18
10. Dependence of DFTB3-D3/3OB results on the target volume used in CSP calculations………………………………………………………………………..S19
11. Structural and energetic information for the 50 most favorable predicted structures of each molecule……………………………………………………………………...S20
12. Supporting References……………………………………………………………….....S21

1. Crystal structure prediction (CSP) sampling convergence

The sampling convergence of the CSP calculations is demonstrated by choosing pyrene-4,5,9,10-tetrone (PTO) as an example. The occurrence of the lowest energy structures in the early PSO generations and re-occurrence of the same structures in later PSO generations illustrates sampling convergence.

As shown in Table S1, for PTO, the lowest energy structure (1) was generated in the 6 generation. The second lowest energy structure (3-6, 9) was first generated in the 5th generation and re-generated in the 31st and 35th generations. The third lowest energy structure (16-30) was first generated in the 4th generation and then re-generated in multiple subsequent generations.

Table S1. CSP results for PTO

Structure index^{a}	Overall No.^{b}	Generation	ΔE_{DFTB3} (kcal/mol)	ΔE_{PBE} (kcal/mol)	Space group
1	27	6	0.0	0.0	$P2_1/c$
3	24	5	0.2	1.1	$P2_1/c$
4	152	31	0.2	1.0	$P2_1/c$
5	174	35	0.2	1.0	$P2_1/c$
6	175	35	0.2	1.0	$P2_1/c$
9	21	5	0.7	1.0	$P2_1/c$
16	17	4	1.3	1.6	$P2_1/c$
17	44	9	1.3	1.6	$P2_1/c$
18	68	14	1.3	1.6	$P2_1/c$
19	72	15	1.3	1.6	$P2_1/c$
20	103	21	1.3	1.6	$P2_1/c$
21	109	22	1.3	1.6	$P2_1/c$
22	110	22	1.3	1.6	$P2_1/c$
23	135	27	1.3	1.6	$P2_1/c$
24	247	50	1.3	1.6	$P2_1/c$
25	237	48	1.3	1.6	$P2_1/c$
26	85	17	1.3	1.6	$P2_1/c$
27	146	30	1.3	1.6	$P2_1/c$
28	196	40	1.3	1.6	$P2_1/c$
29	248	50	1.3	1.6	$P2_1/c$
30	141	29	1.3	1.6	$P2_1/c$

^{a} Sequential number of the structure in the 50 lowest energy DFTB3-D3/3OB structures
^{b} Sequential number of the structure in the 250 structures generated by 50 generations of CSP

The reproducibility of the CSP calculations was verified by running an independent CSP calculation on the test case molecule, PTO. This calculation was also able to find the experimental structure among the four lowest energy structures ranked by DFTB. The 50 lowest energy DFTB structures from this independent calculation have been deposited in the Google Drive repository that is linked in the SI.

2. *k*-grids for each molecule

Table S2 outlines the *k*-grids employed for the geometry and lattice optimizations of the different molecules using PBE-D3/PAW. The number of *k* points along any dimension was calculated by multiplying the reciprocal of the corresponding lattice parameter (*a, b,* or *c*) by a factor of three. The factor of three was determined based on our previous *k*-grid convergence studies for PTO and BQ molecules.[1]

Table S2. Monkhorst-Pack uniform *k*-grids for the different molecules studied

Interaction Type	Molecule	Crystal lattice	Space group	No. of structures	*k*-grid
H- bonding	Ammonia	Cubic	$P2_13$	7	6 x 6 x 6
		Orthorhombic	$P2_12_12_1$	4	10 x 6 x 6
			Pbnm	3	6 x 6 x 10
			$Pca2_1$	5	6 x 10 x 6
		Monoclinic	$P2_1/c$	5	10 x 6 x 6
			Pc	5	6 x 10 x 6
	Acetic acid	Orthorhombic	*Pnma*	13	3 x 4 x 3
		Monoclinic	$P2_1/c$	2	5 x 9 x 3
				1	12 x 3 x 7
				2	13 x 3 x 8
Mixed bonding	Cytosine	Orthorhombic	$P2_12_12_1$	4	13 x 5 x 3
	Imidazole	Orthorhombic	$Pna2_1$	4	3 x 6 x 3
		Monoclinic	$P2_1/c$	4	3 x 6 x 3
vdW	Adamantane	Tetragonal	$P\bar{4}2_1c$	50	4 x 4 x 3
	Benzene	Orthorhombic	*Pbca*	16	4 x 4 x 3
		Tetragonal	$P4_32_12$	21	8 x 8 x 3
		Monoclinic	$P2_1/c$	2	4 x 4 x 3
	CO_2	Cubic	$Pa\bar{3}$	6	6 x 6 x 6
		Orthorhombic	$Pca2_1$	4	6 x 9 x 6
	Benzoquinone (BQ)	Monoclinic	$P2_1/c$	6	10 x 6 x 3
				1	10 x 3 x 5
				3	4 x 3 x 3
	pyrene-4,5,9,10-tetrone (PTO)	Monoclinic	$P2_1/c$	1	14 x 3 x 5
				5	5 x 3 x 3
				2	8 x 4 x 3
				15	15 x 5 x 3

3. Speed tests

To benchmark the difference in computational efficiency between the DFTB3-D3/3OB and PBE-D3/PAW methods, DFTB-predicted crystal structures for some molecules, which were not among the 50 most favorable structures for those molecules, were selected. Single point and optimization calculations were performed on these structures with the DFTB3 and PBE methods. Eight and ten processors were used for the DFTB3 and PBE calculations, respectively, based on their scaling

performance. For both kinds of calculations, an entire 20-processor node was reserved to eliminate interference from other calculations running on the same node and to maximize memory availability. The single point calculations with PBE were slower by a factor of approximately 50-200, while the optimization calculations with PBE were slower by a factor of approximately 3-5 (Table S3). Due to the differing optimization routines and optimization convergence criteria in DFTB+ and Quantum Espresso, used for the DFTB3 and PBE calculations, respectively, the difference in speed between DFTB3 and PBE in the optimization calculations does not correctly reflect the inherent speed difference between these methods. However, based on this limited speed benchmark, using DFTB3 for CSP is still expected to reduce the cost of each CSP generation approximately 3-5 times. This is a significant reduction in computational time since CSP with DFT alone can take up to a few weeks for a molecule like cytosine.

Table S3. CPU time in seconds using DFTB3-D3/3OB and PBE-D3/PAW

	Single point calculation		Geometry and lattice optimization	
	DFTB3[a]	PBE	DFTB3[a]	PBE
Adamantane	1.48	230	4144	22440
Benzene	1.80	85	2253	7597
Cytosine	2.76	478	9707	41460

[a] DFTB3 calculation done using PBE *k*-points.

Table S4 shows that following the DFTB/CALYPSO CSP, the geometry and lattice optimization of the predicted structures with PBE-D3/PAW takes less than or approximately an hour per crystal structure for most molecules, and about 4-5 hours for some molecules. The reoptimization can be carried out simultaneously for several candidate crystal structures. Hence, the PBE post-processing time needed for re-ranking of the predicted structures is not prohibitive.

Table S4. Average CPU time for PBE-D3/PAW optimization of DFTB-predicted crystal structures of the molecules studied.

Interaction Type	Molecule	CPU time (min.)[a]
H – Bonding	Ammonia	27
	Acetic acid	62
Mixed Bonding	Cytosine	305
	Imidazole	89
vdW	Adamantane	28
	Benzene	51
	Carbon dioxide	12
	1,4-benzoquinone	42
	Pyrene-4,5,9,10-tetrone	228

[a] The average CPU time was calculated from the CPU time for PBE optimization of the lowest energy 50 crystal structures predicted by DFTB3-D3/3OB.

4. Finding identical crystal structures

	0	1	2	3	4	5	6
0: AMN.cif	0.0000	0.0226	0.0095	0.0074	0.0026	0.0052	0.0109
1: AMN01.cif	0.0226	0.0000	0.0130	0.0166	0.0217	0.0279	0.0117
2: AMN02.cif	0.0095	0.0130	0.0000	0.0049	0.0088	0.0147	0.0016
3: AMN03.cif	0.0074	0.0166	0.0049	0.0000	0.0056	0.0121	0.0063
4: AMN04.cif	0.0026	0.0217	0.0088	0.0056	0.0000	0.0066	0.0102
5: AMN05.cif	0.0052	0.0279	0.0147	0.0121	0.0066	0.0000	0.0161
6: AMN06.cif	0.0109	0.0117	0.0016	0.0063	0.0102	0.0161	0.0000

Figure S1. Similarity matrix showing the calculated packing similarity of seven predicted crystal structures of ammonia (phase I, $P2_13$). The numbers in the green boxes indicate the $PS_{a,b}$ values.

5. CSP results for carbon dioxide and benzoquinone

(i) Carbon dioxide

Solid CO_2 exists in different phases depending on temperature and pressure. Phase I has a face centered cubic structure ($Pa\bar{3}$ space group) with four molecules per unit cell, a = 5.624 Å at 150 K and ambient pressure (Fig. S2a).[2] Phase II has a body-centered tetragonal structure ($P4_2/mnm$ space group) with two molecules per unit cell, a = 3.535 Å, and c = 4.140 Å at 28 GPa and 680 K (Fig. S2b).[3] Phase III has an orthorhombic structure (*Cmca* space group) with four molecules per unit cell above 11 GPa.[3] Lastly, phase V has an orthorhombic structure ($P2_12_12_1$ space group) with eight molecules per unit cell, a = 6.216 Å, b = 4.352 Å and c = 6.066 Å at 44 GPa.[3, 4]

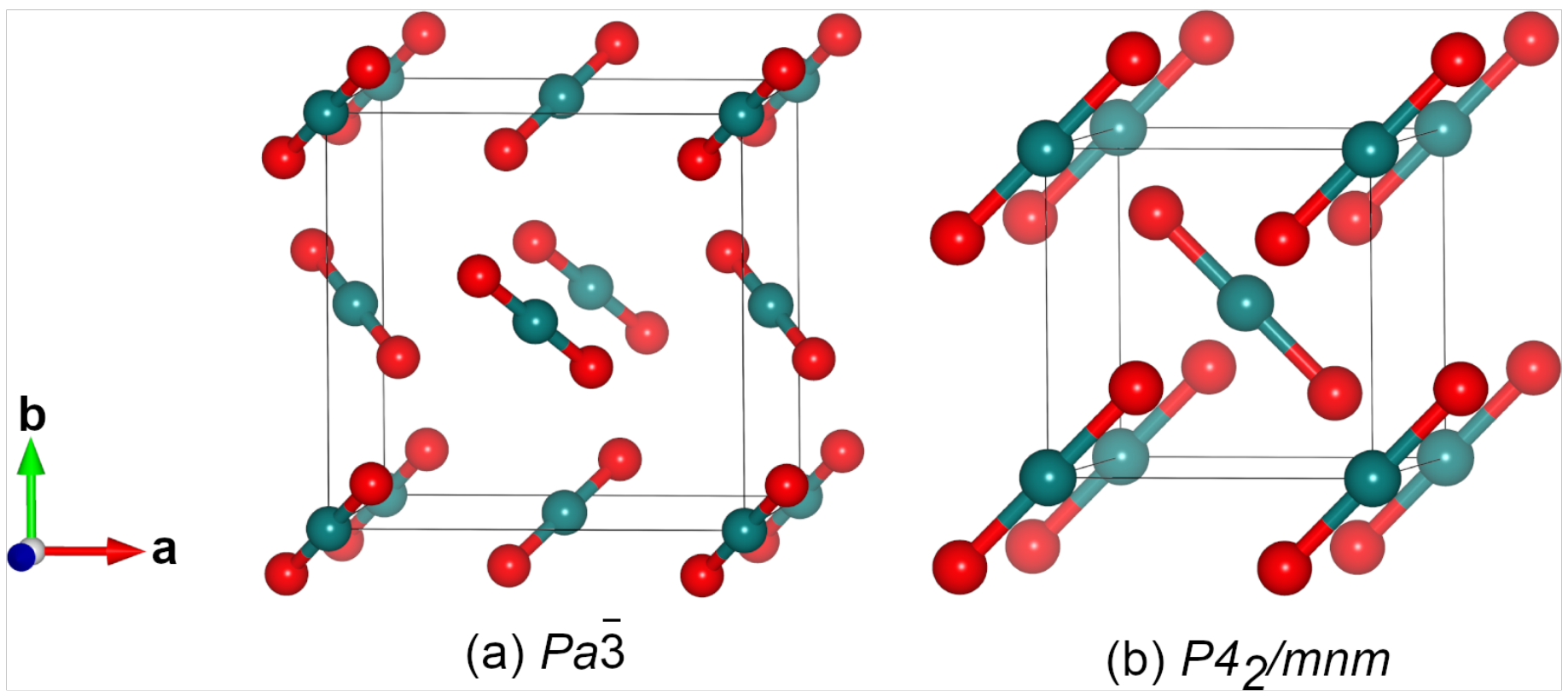

Figure S2. Experimental crystal structures of solid CO_2. (a) Phase I ($Pa\bar{3}$), ICSD Ref. code 16428, (b) Phase II ($P4_2/mnm$), CSD Ref. Code SACBAA. Cyan and red spheres represent C and O atoms, respectively.

Computations identified the most stable polymorph as that belonging to the $Pa\bar{3}$ space group and similar to the experimental phase I (Fig. S3a), with a = 5.624 Å and a = 5.680 Å for the experimental and predicted structures, respectively. We were unable to predict the experimental phases II, III, and V, which are high pressure phases. An orthorhombic crystal structure with four molecules per unit cell was ranked as the second most favorable polymorph (Fig. S3b), followed by a tetragonal structure ($P4_2/mnm$).

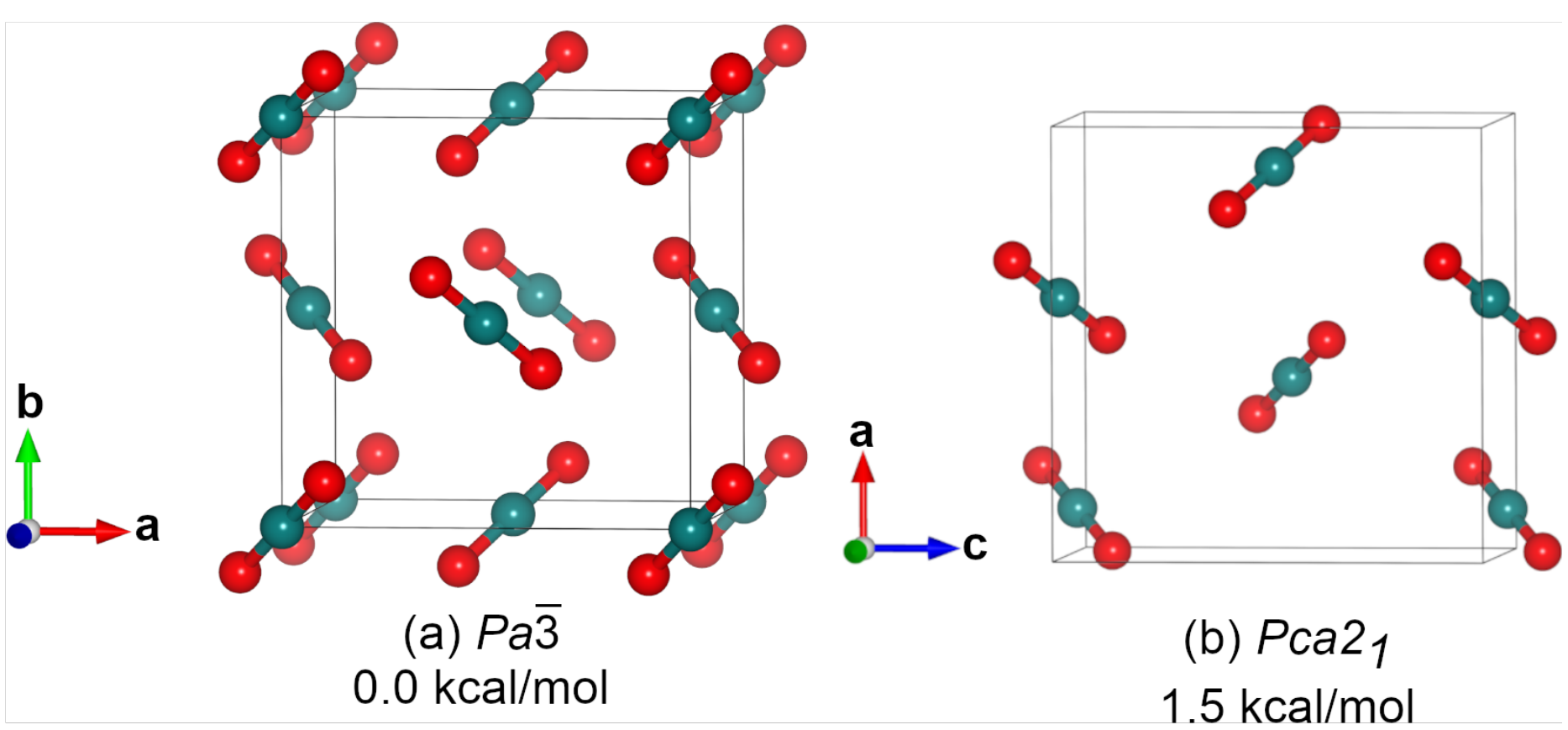

Figure S3. Predicted crystal structures of solid CO_2. (a) Phase I ($Pa\bar{3}$), (b) $Pca2_1$. The PBE energy relative to the most stable crystal structure is shown below each crystal structure.

(ii) 1,4-Benzoquinone (BQ)

There are three experimental crystal structures for BQ reported in the CCDC. These structures have a monoclinic lattice ($P2_1/c$ space group) with two molecules per unit cell and slightly different lattice parameters. The $PS_{a,b}$ value of 0.05 – 0.15 indicates that these structures are identical. For our analysis, the structure with CSD code BNZQUI03 was used. Its lattice parameters are a = 5.743 Å, b = 6.771 Å, c = 6.867 Å, β = 100.14°, V = 262.86 Å^3. Fig. S4 shows the π–stacking interactions between the BQ molecules in this structure.[5, 6]

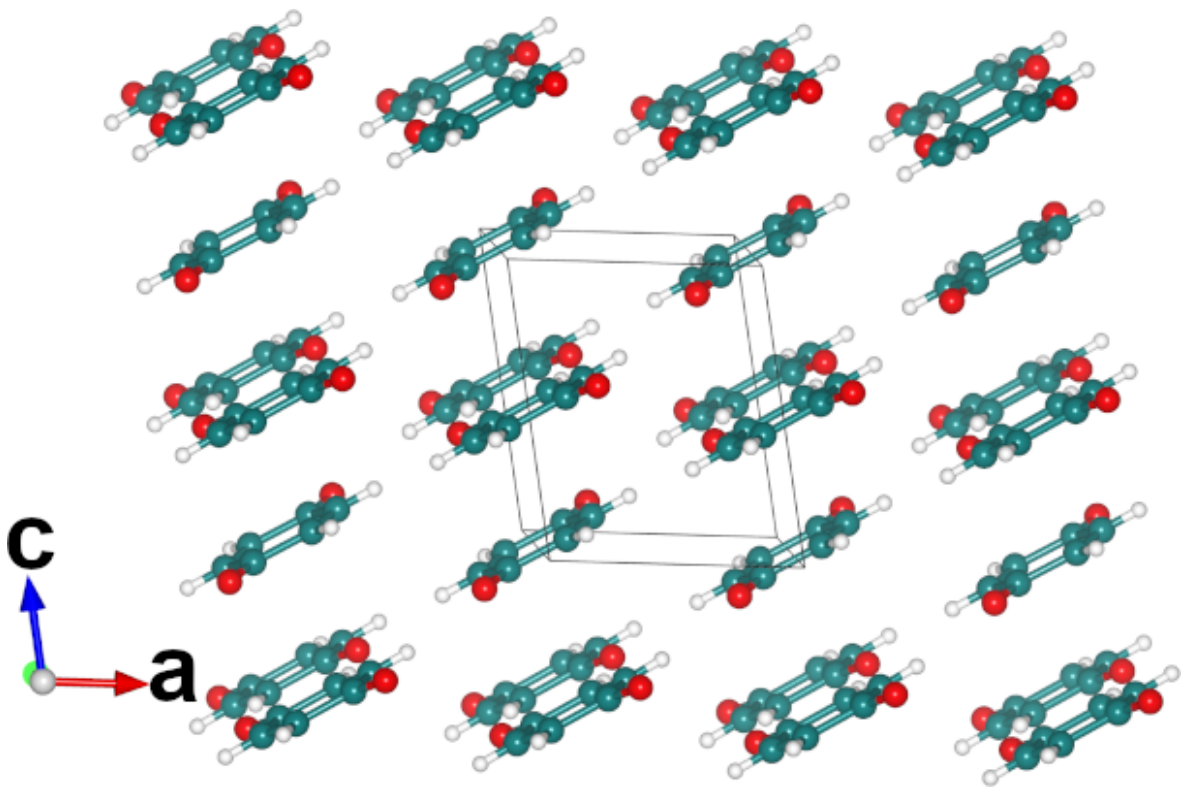

Figure S4. Experimental structure of 1,4-benzoquinone ($P2_1/c$), CSD code BNZQUI03. White, cyan, and red spheres represent H, C, and O atoms, respectively.

Computations predict a monoclinic polymorph (Fig. S5c) that is in agreement with the experimental crystal structure. Additional monoclinic (Fig. S5b) and triclinic (Fig. S5d) polymorphs are also predicted which have similar stability to the one that agrees with experiments (Fig. S5c), all within 0.3 kcal/mol of each other (Tables S18, S19). The monoclinic polymorph shown in Fig. S5a was found to be ~1 kcal/mol higher in energy than the others.

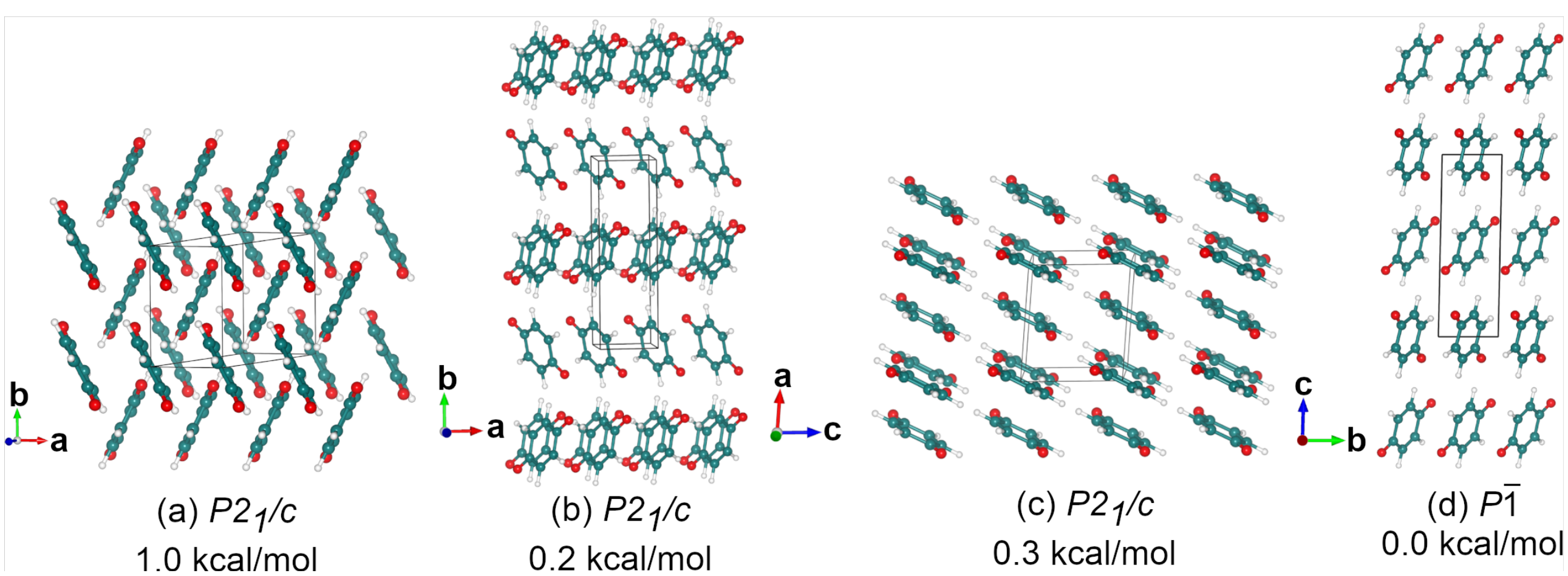

Figure S5. Predicted crystal structures of 1,4-benzoquinone. (a) Structure 12 ($P2_1/c$), (b) structure 30 ($P2_1/c$), (c) structure 32 ($P2_1/c$), and (d) structure 34 ($P\bar{1}$). The PBE energy relative to the most stable crystal structure is shown below each crystal structure.

6. Relative energies and lattice parameters for various polymorphs of the studied molecules

Table S5. Relative energies in kcal/mol for the predicted NH_3 polymorphs

Space group	Crystal Structures[a]	ΔE_{DFTB3}	DFTB3 ranking	ΔE_{PBE}	PBE ranking
$P2_13$	1 – 7	0.0	1	0.0	1
$P2_12_12_1$	8 – 11	0.6	2	2.5 – 2.6	6
Pbnm	12 – 14	0.6	2	1.7 – 1.8	5
$P2_1/c$	15 – 17, 20 – 21	1.5 – 1.7	4	1.0 – 1.1	4
$Pca2_1$	32 – 36	2.7	5	0.8	3
Pc	39 – 43	3.1	6	0.3 – 0.4	2

[a] The crystal structures are numbered according to their DFTB3 energy, with crystal structure 1 having the lowest DFTB3 energy.

Table S6. Optimized lattice parameters for the predicted crystal structures of NH_3 using PBE

Space group	Lattice Parameter	Expt.	PBE
$P2_13$	*a* (Å)	5.048[a]	4.963
	b (Å)	5.048[a]	4.963
	c (Å)	5.048[a]	4.963
$P2_12_12_1$	*a* (Å)	3.250[b]	3.508
	b (Å)	5.658[b]	5.743
	c (Å)	5.356[b]	5.784
Pc	*a* (Å)	-	6.571
	b (Å)	-	3.292
	c (Å)	-	6.181
	β (°)	-	115.20
$Pca2_1$	*a* (Å)	-	6.094
	b (Å)	-	3.292
	c (Å)	-	6.024
$P2_1/c$	*a* (Å)	-	3.307
	b (Å)	-	6.102
	c (Å)	-	6.017
	β (°)	-	97.68
Pbmn	*a* (Å)	-	5.797
	b (Å)	-	5.119
	c (Å)	-	4.051

[a] Ref.[7] (0.00 GPa, 2K, ICSD Ref. Code 49697), [b] Ref.[8] (9.00 GPa, ICSD Ref. Code 83234)

Table S7. Relative energies in kcal/mol for the predicted acetic acid polymorphs

Space group	Crystal Structures[a]	ΔE_{DFTB3}	DFTB3 ranking	ΔE_{PBE}	PBE ranking
$P2_1/c$	1-2	0.0	1	2.4 – 2.6	4
Pnma	7 -19	1.0	2	2.3	3
$P2_1/c$	20	1.1	3	0.0	1
$P2_1/c$	43 - 44	2.4	4	2.1	2

[a] The crystal structures are numbered according to their DFTB3 energy, with crystal structure 1 having the lowest DFTB3 energy.

Table S8. Optimized lattice parameters for the predicted crystal structures of acetic acid using PBE

Space group	Crystal Structure	Lattice parameter	Expt.	PBE
$P2_1/c$	20	*a* (Å)	3.945[a]	3.949
		b (Å)	13.028[a]	13.110
		c (Å)	5.641[a]	6.741
		β (°)	93.18[a]	123.03
		V (Å^3)	289.44[a]	292.57
$P2_1/c$	1	*a* (Å)	-	7.048
		b (Å)	-	4.420
		c (Å)	-	10.216
		β (°)	-	102.49
		V (Å^3)	-	310.73
Pnma	7	*a* (Å)	-	7.002
		b (Å)	-	6.476
		c (Å)	-	7.019
$P2_1/c$	43	*a* (Å)	-	4.242
		b (Å)	-	13.475
		c (Å)	-	5.511
		β (°)	-	94.88
		V (Å^3)	-	313.89

[a] CSD code ACETAC09

Table S9. Optimized lattice parameters for the predicted crystal structures of cytosine using PBE

Space group	Crystal Structure	Lattice Parameter	Expt.[a]	PBE
$P2_12_12_1$	1	*a* (Å)	13.044	12.983
		b (Å)	9.496	9.445
		c (Å)	3.814	3.778

[a] CSD code CYSTIN01.

Table S10. Mean experimental lattice parameters of the monoclinic ($P2_1/c$ *or* $P2_1/n$) crystal structures of imidazole[a]

Group	Lattice parameters				
	a (Å)	*b* (Å)	*c* (Å)	β (°)	*V* (Å^3)
1[b]	7.651 (0.07)	5.402 (0.04)	9.574 (0.32)	109.76 (17.63)	355.74 (7.93)
2[c]	7.507 (0.07)	5.204 (0.08)	9.687 (0.04)	120.49 (0.83)	326.14 (11.99)
3[d]	7.367 (0.04)	5.041 (0.05)	9.583 (0.03)	122.23 (0.47)	301.07 (7.18)

[a] Numbers in parentheses represent standard deviation.
[b] Structures with CSD code IMAZOL01, IMAZOL02, IMAZOL03, IMAZOL04, IMAZOL06, IMAZOL10, IMAZOL13, IMAZOL14, IMAZOL15, and IMAZOL34.
[c] Structures with CSD code IMAZOL16, IMAZOL17, and IMAZOL18.
[d] Structures with CSD code IMAZOL19, IMAZOL20, and IMAZOL21.

Table S11. Mean experimental lattice parameters of the orthorhombic (*Aea*2) crystal structures of imidazole[a]

Lattice parameters			
a (Å)	*b* (Å)	*c* (Å)	*V* (Å^3)
13.947 (0.18)	8.635 (0.03)	5.321 (0.05)	640.85 (13.38)

[a] Numbers in parentheses represent standard deviation. Structures with CSD code IMAZOL22, IMAZOL23, IMAZOL24, IMAZOL25, and IMAZOL26.

Table S12. Relative energies in kcal/mol for the predicted imidazole polymorphs

Space Group	Crystal Structures[a]	ΔE_{DFTB3}	DFTB3 ranking	ΔE_{PBE}	PBE ranking
$Pna2_1$	1-3	0.0	1	2.7	2
$P2_1/c$	14	0.6	2	0.0	1

[a] The crystal structures are numbered according to their DFTB3 energy, with crystal structure 1 having the lowest DFTB3 energy.

Table S13. Optimized lattice parameters for the predicted crystal structures of imidazole using PBE

Space group	Crystal Structure	Lattice Parameter	Expt.	PBE
$P2_1/c$	14	*a* (Å)	7.651 (0.07)[a]	7.602
		b (Å)	5.402 (0.04)[a]	5.274
		c (Å)	9.574 (0.32)[a]	9.726
		β (°)	109.76 (17.63)[a]	116.80
		V (Å^3)	355.74 (7.93)[a]	348.02
$Pna2_1$	1	*a* (Å)	-	9.298
		b (Å)	-	4.417
		c (Å)	-	8.693

[a] Experimental values from Table S10, group 1.

Table S14. Mean experimental lattice parameters of the orthorhombic (*Pbca*) crystal structures of benzene [a]

Group	Lattice parameters			
	a (Å)	*b* (Å)	*c* (Å)	*V* ($Å^3$)
1[b]	7.240 (0.2)	8.805 (1.04)	7.753 (1.40)	484.83 (10.63)
2[c]	7.381 (0.03)	9.409 (0.04)	6.740 (0.05)	468.09 (6.93)
3[d]	7.250 (0.03)	9.187 (0.13)	6.678 (0.08)	444.87 (12.82)
4[e]	7.244 (0.31)	8.570 (1.54)	8.350 (1.87)	507.41 (1.13)

[a] Numbers in parentheses represent standard deviation.
[b] Structures with CSD code BENZEN, BENZEN01, BENZEN15, BENZEN18, BENZEN19, BENZEN25.
[c] Structures with CSD code BENZEN06, BENZEN07, BENZEN14, BENZEN20.
[d] Structures with CSD code BENZEN11, BENZEN12, BENZEN13.
[e] Structures with CSD code BENZEN02, BENZEN26.

Table S15. Mean experimental lattice parameters of the monoclinic ($P2_1/c$) crystal structures of benzene [a]

Group	Lattice parameters				
	a (Å)	*b* (Å)	*c* (Å)	β (°)	*V* ($Å^3$)
5[b]	5.417	5.376	7.532	110.00	206.12
6[c]	5.518 (0.01)	5.467 (0.04)	7.663 (0.01)	110.57 (0.03)	216. 46 (0.93)
7[d]	5.224 (0.13)	5.218 (0.12)	7.223 (0.18)	109.3 (0.73)	186.0 (12.46)

[a] Numbers in parentheses represent standard deviation.
[b] CSD code BENZEN03.
[c] Structures with CSD code BENZEN16, BENZEN17.
[d] Structures with CSD code BENZEN21, BENZEN23, BENZEN24.

Table S16. Relative energies in kcal/mol for the predicted benzene polymorphs

Structure	Space group	ΔE_{DFTB3}	DFTB3 ranking	ΔE_{PBE}	PBE ranking
Monoclinic	$P2_1/c$	0.0	1	1.7	2
Tetragonal	$P4_32_12$	0.1 – 0.2	2	2.2	3
Orthorhombic	*Pbca*	0.2	3	0.0	1

Table S17. Optimized lattice parameters for the predicted crystal structures of benzene using PBE

Space group	Crystal Structure	Lattice Parameter	Expt.	PBE
Pbca	Orthorhombic	*a* (Å)	7.381 (0.03)[a]	7.346
		b (Å)	9.409 (0.04)[a]	9.219
		c (Å)	6.740 (0.05)[a]	6.808
$P4_32_12$	Tetragonal	*a* (Å)	-	5.611
		b (Å)	-	5.611
		c (Å)	-	14.484
$P2_1/c$	Monoclinic	*a* (Å)	5.518 (0.01)[b]	5.644
		b (Å)	5.467 (0.04)[b]	5.557
		c (Å)	7.663 (0.01)[b]	7.768
		β (°)	110.57 (0.04)[b]	110.41
		V (Å^3)	216.46 (0.93)[b]	228.36

[a] Experimental values were obtained from Table S14, group 2.
[b] Experimental values were obtained from Table S15, group 6.

Table S18. Relative energies in kcal/mol for the predicted BQ polymorphs

Crystal Structures[a]	Space group	ΔE_{DFTB3}	DFTB3 ranking	ΔE_{PBE}	PBE ranking
12-17	$P2_1/c$	0.4	2	1.0	4
30		1.9	3	0.2	2
29, 32-33		1.9	3	0.3	3
34	$P\bar{1}$	2.0	4	0.0	1

[a] The crystal structures are numbered according to their DFTB3 energy, with three polymeric structures having the lowest DFTB3 energy (not listed here).

Table S19. Optimized lattice parameters for the predicted crystal structures of BQ using PBE

Space Group	Crystal Structure	Lattice Parameter	Expt.[a]	PBE
$P2_1/c$	29	a (Å)	5.743[a]	5.673
		b (Å)	6.771[a]	6.637
		c (Å)	6.867[a]	6.868
		α (°)	90.00[a]	89.21
		β (°)	100.14[a]	81.63
		γ (°)	90.00[a]	89.42
		V (Å^3)	262.86[a]	255.86
$P2_1/c$	32 – 33	a (Å)	5.743[a]	5.678
		b (Å)	6.771[a]	6.637
		c (Å)	6.867[a]	6.868
		α (°)	90.00[a]	90.00
		β (°)	100.14[a]	98.43
		γ (°)	90.00[a]	90.00
		V (Å^3)	262.86[a]	256.01
$P2_1/c$	12 – 17	a (Å)	-	3.654
		b (Å)	-	5.802
		c (Å)	-	11.435
		β (°)	-	93.26
		V (Å^3)	-	241.99
$P2_1/c$	30	a (Å)	-	3.664
		b (Å)	-	11.700
		c (Å)	-	5.947
		β (°)	-	96.54
		V (Å^3)	-	253.29
$P\bar{1}$	34	a (Å)	-	5.853
		b (Å)	-	3.769
		c (Å)	-	11.550
		α (°)	-	88.07
		β (°)	-	81.88
		γ (°)	-	84.94
		V (Å^3)	-	251.17

[a] CSD code BNZQUI03

Table S20. Relative energies in kcal/mol for the predicted PTO polymorphs belonging to the *P*21/*c* space group

Space Group	Crystal Structures[a]	ΔE_{DFTB3}	DFTB3 ranking	ΔE_{PBE}	PBE ranking
$P2_1/c$	1	0.0	1	0.0	1
	3 – 6, 9	0.2	2	1.0 – 1.1	2
	7-8	0.5	3	2.5	4
	16 – 30	1.3 – 1.5	4	1.6 – 1.7	3

[a] The crystal structures are numbered according to their DFTB3 energy, with crystal structure 1 having the lowest DFTB3 energy.

Table S21. Optimized lattice parameters for the predicted crystal structures of PTO using PBE.

Space group	Crystal Structure	Lattice Parameter	Expt.	PBE
$P2_1/c$	1	*a* (Å)	3.728	3.781
		b (Å)	15.405	15.271
		c (Å)	9.243	9.175
		β (°)	100.45	98.87
		V (Å^3)	522.08	523.45
	3	*a* (Å)	-	6.250
		b (Å)	-	9.071
		c (Å)	-	9.683
		β (°)	-	105.59
		V (Å^3)	-	528.73
	7	*a* (Å)	-	4.611
		b (Å)	-	9.451
		c (Å)	-	12.299
		β (°)	-	102.50
		V (Å^3)	-	523.27
	16	*a* (Å)	-	3.820
		b (Å)	-	8.880
		c (Å)	-	15.682
		β (°)	-	94.00
		V (Å^3)	-	530.69

7. Experimental crystal structure of pyrene-4,5,9,10-tetrone (PTO)

Table S22. Atomic coordinates ($\times 10^4$) and equivalent isotropic displacement parameters ($Å^2 \times 10^3$) for PTO. U(eq) is defined as one third of the trace of the orthogonalized U^{ij} tensor.

	x	y	z	U(eq)
O(1)	1101(6)	6077(1)	1335(2)	56(1)
C(1)	2023(8)	5799(2)	2573(3)	48(1)
O(2)	11038(6)	3076(1)	6379(2)	55(1)
C(2)	3920(8)	4964(2)	2916(3)	46(1)
C(6)	7134(8)	3923(2)	4623(3)	46(1)
C(3)	4347(8)	4414(2)	1758(3)	48(1)
C(8)	8869(8)	3662(2)	6124(3)	46(1)
C(7)	5245(8)	4715(2)	4378(3)	44(1)
C(4)	6092(9)	3630(2)	2014(3)	51(1)
C(5)	7516(8)	3386(2)	3443(3)	48(1)

Table S23. Bond lengths (Å) and angles (°) for PTO

O(1)-C(1)	1.211(3)
C(1)-C(2)	1.475(5)
C(1)-C(8)#1	1.547(4)
O(2)-C(8)	1.207(4)
C(2)-C(3)	1.396(4)
C(2)-C(7)	1.405(4)
C(6)-C(5)	1.396(4)
C(6)-C(7)	1.407(5)
C(6)-C(8)	1.476(4)
C(3)-C(4)	1.372(5)
C(3)-H(3)	0.9500
C(8)-C(1)#1	1.547(4)
C(7)-C(7)#1	1.484(6)
C(4)-C(5)	1.384(4)
C(4)-H(4)	0.9500
C(5)-H(5)	0.9500

O(1)-C(1)-C(2)	123.6(3)
O(1)-C(1)-C(8)#1	118.8(3)
C(2)-C(1)-C(8)#1	117.6(3)
C(3)-C(2)-C(7)	120.2(3)
C(3)-C(2)-C(1)	118.8(3)
C(7)-C(2)-C(1)	121.0(3)
C(5)-C(6)-C(7)	120.5(3)
C(5)-C(6)-C(8)	118.9(3)
C(7)-C(6)-C(8)	120.6(3)
C(4)-C(3)-C(2)	121.2(3)
C(4)-C(3)-H(3)	119.4
C(2)-C(3)-H(3)	119.4
O(2)-C(8)-C(6)	123.5(3)
O(2)-C(8)-C(1)#1	118.7(3)
C(6)-C(8)-C(1)#1	117.8(3)
C(2)-C(7)-C(6)	118.0(3)
C(2)-C(7)-C(7)#1	120.8(3)
C(6)-C(7)-C(7)#1	121.1(3)
C(3)-C(4)-C(5)	119.4(3)
C(3)-C(4)-H(4)	120.3
C(5)-C(4)-H(4)	120.3
C(4)-C(5)-C(6)	120.6(3)
C(4)-C(5)-H(5)	119.7
C(6)-C(5)-H(5)	119.7

Symmetry transformations used to generate equivalent atoms:
#1 -x+1,-y+1,-z+1

Table S24. Anisotropic displacement parameters (Å^2×10^3) for PTO. The anisotropic displacement factor exponent takes the form: $-2\pi^2[h^2a^{*2}U^{11} + ... + 2 h k a^* b^* U^{12}]$

	U^{11}	U^{22}	U^{33}	U^{23}	U^{13}	U^{12}
O(1)	79(2)	61(2)	28(1)	6(1)	10(1)	2(1)
C(1)	59(2)	55(2)	32(2)	2(1)	12(1)	-4(1)
O(2)	69(1)	55(1)	41(1)	4(1)	17(1)	5(1)
C(2)	55(2)	53(2)	31(2)	2(1)	15(1)	-3(1)
C(6)	56(2)	52(2)	33(2)	1(1)	16(1)	-4(1)
C(3)	60(2)	58(2)	29(1)	1(1)	14(1)	-4(1)
C(8)	56(2)	49(2)	36(2)	3(1)	15(1)	-2(1)
C(7)	54(2)	52(2)	30(2)	0(1)	15(1)	-4(1)
C(4)	65(2)	59(2)	34(2)	-6(1)	21(1)	-6(1)
C(5)	60(2)	49(2)	39(2)	2(1)	19(1)	-2(1)

Table S25. Hydrogen coordinates (×10^4) and isotropic displacement parameters (Å^2×10^3) for PTO.

	x	y	z	U(eq)
H(3)	3413	4586	775	58
H(4)	6320	3257	1216	61
H(5)	8765	2849	3624	58

8. Average unit cell volume of DFTB3-predicted structures

Table S26. Average unit cell volumes for different molecules as predicted by DFTB3-D3/3OB

Interaction Type	Molecule	Target Volume (Å^3)^a	Average Volume (Å^3)^b
H – bonding	Ammonia	125.00	88.82 (2.29)
	Acetic Acid	290.00	260.12 (7.19)
Mixed bonding	Cytosine	470.00	440.61 (13.20)
	Imidazole	300.00	308.90 (4.33)
vdW	Adamantane	370.00	349.43 (0.16)
	Benzene	450.00	404.19 (5.07)
	CO_2	185.00	160.09 (2.62)
	BQ	250.00	217.51 (5.99)
	PTO	500.00	479.50 (7.73)

^{a} The target unit cell volume was chosen to be close to the experimental unit cell volume.
^{b} The average volume was calculated from the lowest energy 50 crystal structures predicted by DFTB3-D3/3OB. The numbers in parentheses represent standard deviations. Structures with volumes above or ~100Å^3 below the target volume were not included in the mean/standard deviation calculation.

9. Calculation of target volume in the absence of experimental crystal structure

In the absence of experimental unit cell volume data, the target volume for CSP can be approximated using the shape of the molecule, the type of interaction between the molecules, and the experimentally known number of molecules per unit cell. The shape of most of the molecules studied here can be assumed to be cylindrical, for example, benzene, benzoquinone, CO_2, pyrene-4,5,9,10-tetrone (PTO), ammonia, cytosine, and imidazole. Adamantane and acetic acid can be assumed to be a sphere and a cuboid, respectively. The volume of a single molecule, based on its assumed geometrical shape, can be multiplied by the number of molecules per unit cell to obtain the target unit cell volume. The procedure used to obtain the dimensions of the geometrical shapes corresponding to the different molecules is described here.

For the molecules considered to be cylindrical, with the exception of CO_2 and ammonia, the radius of the inscribed or circumscribed circle, based on the best fit to the molecule, was taken to be the minimum radius of the cylinder. For molecules in the H-bonding class or mixed bonding class, the radius was extended by 0.75-1.0 Å. For molecules in the vdW class, the radius was extended by 1.0 Å. These extensions to the radius account for intermolecular distances in the crystal. The height of the cylinder was taken to be equal to the van der Waals diameter of a carbon atom.

For CO_2, the length of the molecule plus twice the van der Waals radius of oxygen determined the height of the cylinder while the radius was taken to be equal to the van der Waals radius of carbon. For ammonia, the radius of the circle circumscribing the three hydrogen atoms determined the minimum radius of the cylinder. A value of 0.90 Å was added to this radius to account for H-bonding interactions between ammonia molecules. The vertical distance between the N atom and the plane containing the H atoms, with a value of 1.5 Å (0.75 Å x 2) added to it, determined the height of the cylinder.

For adamantane, the radius of the corresponding sphere was calculated as half of the maximum distance between the atoms in the molecule, and a value of 1.0 Å was added to the radius to account for the intermolecular interactions.

For acetic acid, the length of the corresponding cuboid was calculated from the distance between an oxygen atom and a methyl hydrogen atom, and a value of 1.9 Å added to account for the intermolecular hydrogen bonding and van der Waals interactions. The width of the cuboid was calculated as the distance between the two oxygen atoms with an added 2 Å to account for van der Waals interactions. The height was calculated as the distance between any two methyl hydrogen atoms with an added 2 Å for van der Waals interactions.

10. Dependence of DFTB3-D3/3OB results on the target volume used in CSP calculations

A new CSP calculation was performed for the test-case molecule, PTO, with a target unit cell volume of 525 Å^3, as opposed to the value of 500 Å^3 used earlier. Both CSP calculations with the target volume of 500 Å^3 located the experimental crystal structure within the four lowest energy structures ranked by DFTB3-D3/3OB. In contrast, the CSP calculation with the larger target volume of 525 Å^3 failed to locate the experimental crystal structure within the 50 lowest energy structures from DFTB.

As shown in Table S27, the average unit cell volume in all three CSP calculations was ~480 Å^3, with a much larger standard deviation in the CSP calculation with a target unit cell volume of 525 Å^3. Fig. S6 shows that in the latter, the distribution of unit cell volume of the 50 lowest energy structures did not have a clear peak, while in the two CSP calculations with a target unit cell volume of 500 Å^3, most structures had unit cell volumes in the range 470-480 Å^3. The experimental crystal structure has a DFTB unit cell volume of ~470 Å^3, which is not sampled in the CSP calculation with a target unit cell volume of 525 Å^3.

A close look at Table 6 in the main text shows that for most of the molecules, the average of the DFTB volumes of the fifty most favorable structures predicted by DFTB is lower by 30-40 Å^3 than the experimental unit cell volume. CO_2 and benzene are major exceptions to this, with deviation from the experimental volume being ~20 Å^3 for CO_2 and ~70 Å^3 for benzene. The CSP for CO_2 was successful despite using a target unit cell volume larger than the experimental volume. This analysis indicates that especially for molecules with planar aromatic ring structures, the target unit cell volume in the CSP workflow should be set to be ~30 Å^3 lower than the experimental or calculated unit cell volume so that DFTB, which tends to underestimate the unit cell volume, can successfully locate the experimental crystal structure.

Table S27. Influence of target volume in CSP calculations on the average DFTB3-D3/3OB unit cell volumes for PTO

Target Volume (Å^3)	Average Volume^a (Å^3)
500.00, run 1	479.50 (7.73)
500.00, run 2	479.86 (6.48)
525.00	480.61 (11.19)

^{a} The average volume was calculated from the lowest energy 50 crystal structures predicted by DFTB3-D3/3OB. The numbers in parentheses represent standard deviations. Structures with volumes above or ~100 Å^3 below the target volume were not included in the mean/standard deviation calculation.

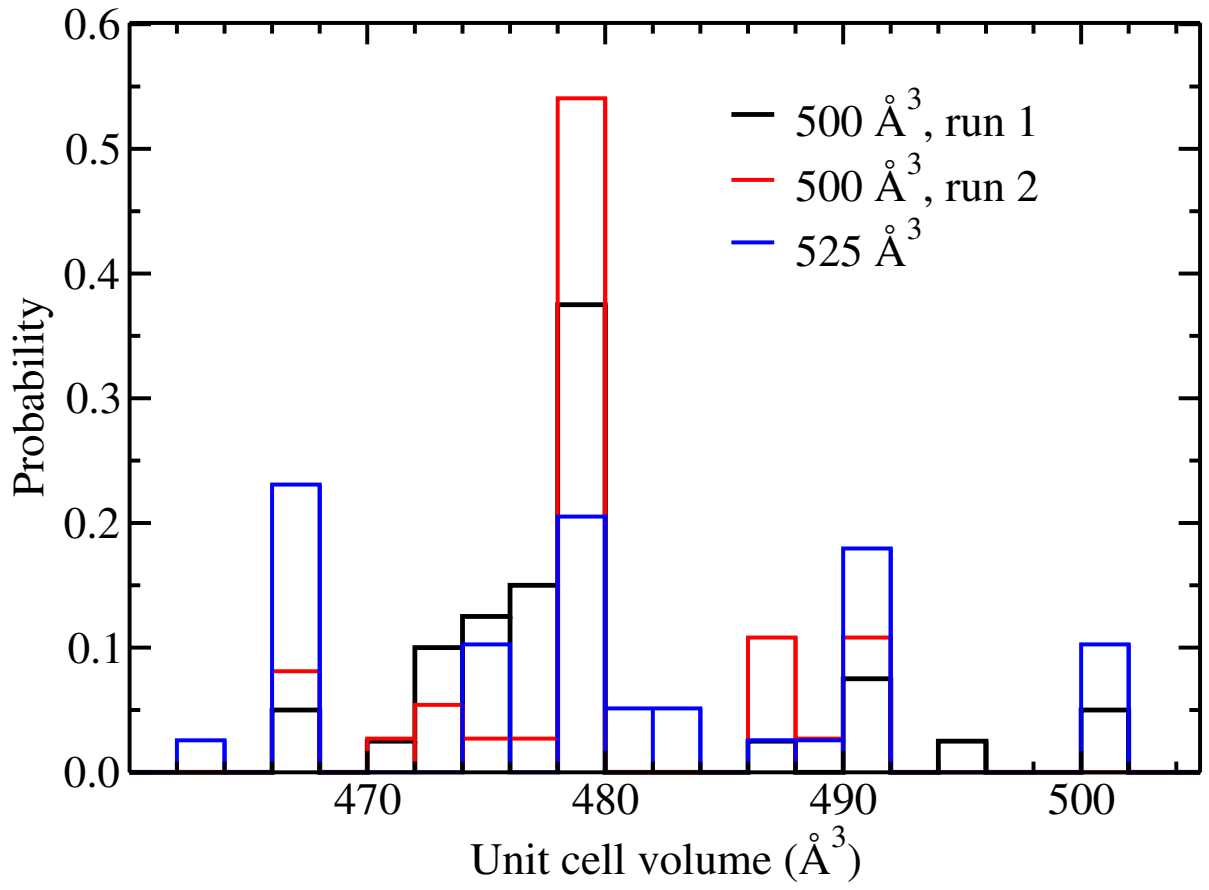

Figure S6. Probability distribution of DFTB3-D3/3OB unit cell volumes in different independent CSP calculations for PTO with target unit cell volumes of 500 Å^3 and 525 Å^3.

11. Structural and energetic information for the 50 most favorable predicted structures of each molecule

Link to Google Drive folder:
https://drive.google.com/drive/folders/1ywThnSK-n3pX7lKglZ7dE53C42iwvqTW?usp=sharing

Supporting References

(1) Kitheka, M. M.; Redington, M.; Zhang, J.; Yao, Y.; Goyal, P. Benchmarks of the Density Functional Tight-Binding Method for Redox, Protonation and Electronic Properties of Quinones. *Phys. Chem. Chem. Phys.* **2022**, *24*, 6742-6756.
(2) Simon, A.; Peters, K. Single-Crystal Refinement of the Structure of Carbon Dioxide. *Acta Cryst. B* **1980**, *36*, 2750-2751.
(3) Yoo, C. S.; Cynn, H.; Gygi, F.; Galli, G.; Iota, V.; Nicol, M.; Carlson, S.; Häusermann, D.; Mailhiot, C. Crystal Structure of Carbon Dioxide at High Pressure: "Superhard" Polymeric Carbon Dioxide. *Phys. Rev. Lett.* **1999**, *83* (26), 5527-5530.
(4) Yong, X.; Liu, H.; wu, M.; Yoo, C.-S. Crystal Structures and Dynamical Properties of Dense CO_2. *Proc. Natl. Acad. Sci. U. S. A.* **2016**, *113*, 11110-11115.
(5) Bolhuis, F. V.; Kiers, C. T. Refinement of the Crystal Structure of *p*-Benzoquinone at -160°C. *Acta Cryst. B* **1978**, *34*, 1015-1016.
(6) Trotter, J. A Three-Dimensional Analysis of the Crystal Structure of *p*-Benzoquinone. *Acta Cryst.* **1960**, *13*, 86.
(7) Hewat, A. W.; Riekel, C. The Crystal Structure of Deuteroammonia between 2 and 180 K by Neutron Powder Profile Refinement. *Acta Cryst. A* **1979**, *35*, 569-571.
(8) Loveday, J. S.; Nelmes, R. J.; Marshall, W. G.; Besson, J. M.; Koltz, S.; Hamel, G. Structure of Deuterated Ammonia IV. *Phys. Rev. Lett.* **1996**, *76* (1), 74-77.
